# Thermophiles and carbohydrate-active enzymes (CAZymes) in biofilm microbial consortia that decompose lignocellulosic plant litters at high temperatures

**DOI:** 10.1038/s41598-022-06943-9

**Published:** 2022-02-18

**Authors:** Kok Jun Liew, Chee Hung Liang, Yee Ting Lau, Amira Suriaty Yaakop, Kok-Gan Chan, Saleha Shahar, Mohd Shahir Shamsir, Kian Mau Goh

**Affiliations:** 1 Faculty of Science, Universiti Teknologi Malaysia, 81310 Skudai, Johor Malaysia; 2 School of Biological Sciences, Universiti Sains Malaysia, 11800, Gelugor, Pulau Pinang Malaysia; 3 Division of Genetics and Molecular Biology, Institute of Biological Sciences, Faculty of Science, University of Malaya, 50603 Kuala Lumpur, Malaysia; 4 Faculty of Applied Sciences and Technology, Universiti Tun Hussein Onn Malaysia, 84600 Pagoh, Johor Malaysia

**Keywords:** Microbial ecology, Biofilms, Bioinformatics, Water microbiology

## Abstract

The SKY hot spring is a unique site filled with a thick layer of plant litter. With the advancement of next-generation sequencing, it is now possible to mine many new biocatalyst sequences. In this study, we aimed to (i) identify the metataxonomic of prokaryotes and eukaryotes in microbial mats using 16S and 18S rRNA markers, (ii) and explore carbohydrate degrading enzymes (CAZymes) that have a high potential for future applications. Green microbial mat, predominantly photosynthetic bacteria, was attached to submerged or floating leaves litter. At the spring head, the sediment mixture consisted of plant debris, predominantly brownish-reddish gelatinous microbial mat, pale tan biofilm, and grey-white filament biofilm. The population in the spring head had a higher percentage of archaea and hyperthermophiles than the green mat. Concurrently, we cataloged nearly 10,000 sequences of CAZymes in both green and brown biofilms using the shotgun metagenomic sequencing approach. These sequences include β-glucosidase, cellulase, xylanase, α-N-arabinofuranosidase, α-l-arabinofuranosidase, and other CAZymes. In conclusion, this work elucidated that SKY is a unique hot spring due to its rich lignocellulosic material, often absent in other hot springs. The data collected from this study serves as a repository of new thermostable macromolecules, in particular families of glycoside hydrolases.

## Introduction

Hot springs are one of the main reservoirs for thermophiles and functional macromolecules^1–4^. Besides the essential factors of temperature and pH, physicochemical parameters, such as salinity, may govern the hot spring microbiota and its genomic and macromolecule content^5–7^. Metagenome data from over 1000 hot springs has been registered in the NCBI database and extensive microbiota research has been conducted for heated springs in Argentina^8^, India^9–14^, Japan^7^, United States^15,16^, Russia^17–19^, New Zealand^20,21^, China^22^, and other countries^23,24^. Microbial diversity in hot springs may vary between the sediment, soil, and water even though these samples were taken from the same hot spring^25^. The scientific community also pays attention to biofilms at heated places^17,18,23,26,27^. Among the earlier publications, only a handful of hot springs contained plant residues, such as Obsidian Pool at Yellowstone Natural Park (USA)^16^, Deulajhari (India)^28^, and Malaysia SKY Hot Springs^29^.

Thermophiles and their thermostable enzymes are essential for white biotechnology applications^1^. Recombinant novel xylanase obtained from a Spanish hot spring fosmid library, is extremely thermostable where it can retain ~ 70% of initial activity after 24 h at 70 °C^4^. Shotgun metagenomics using hot spring samples is an emerging technique to mine massive genes encoding functional macromolecules and bypass conventional routes. In one interesting article, Kaushal et al.^3^ performed shotgun metagenomic of four Indian hot springs (55–98 °C) and generated contigs that contained over 4000 putative genes encoding for carbohydrate-acting enzymes (CAZymes). Recently, Reichart et al.^2^ data-mined CAZymes sequences from 71 hot springs’ shotgun metagenomic datasets.

The breakdown of lignocellulosic polymers requires multiple enzymes generated by the microbial consortium^30^. These enzymes include glycosyl hydrolases (GHs), carbohydrate esterases (CEs), and auxiliary activities enzymes (AAs). The sugars liberated from lignocellulosic polymers can then be fermented to produce biofuels. The majority of CAZymes in hot springs are yet to be discovered for biofuel production^2^. In addition, previous experiments in heated lab setups or in-situ hot springs enriched with insoluble cellulosic biomass, aimed to elucidate the role of thermophiles and thermozymes in lignocellulose degradation^31–33^.

Malaysia has more than 60 known hot springs with temperatures ranging from 36 to 102 °C^29,34^. A few years ago, we encountered SKY hot spring with degrading plant litters and we performed a snapshot on the prokaryotic diversity using amplicon sequencing; yet the functional genes acting on lignocellulosic polymers remain uncertain^29^; therefore we will address this research gap in the current report. This work will provide new enzyme targets for future use for white biotechnology, not limited to biofuel industries.

## Results

### Site and samples description

**Figure 1:**
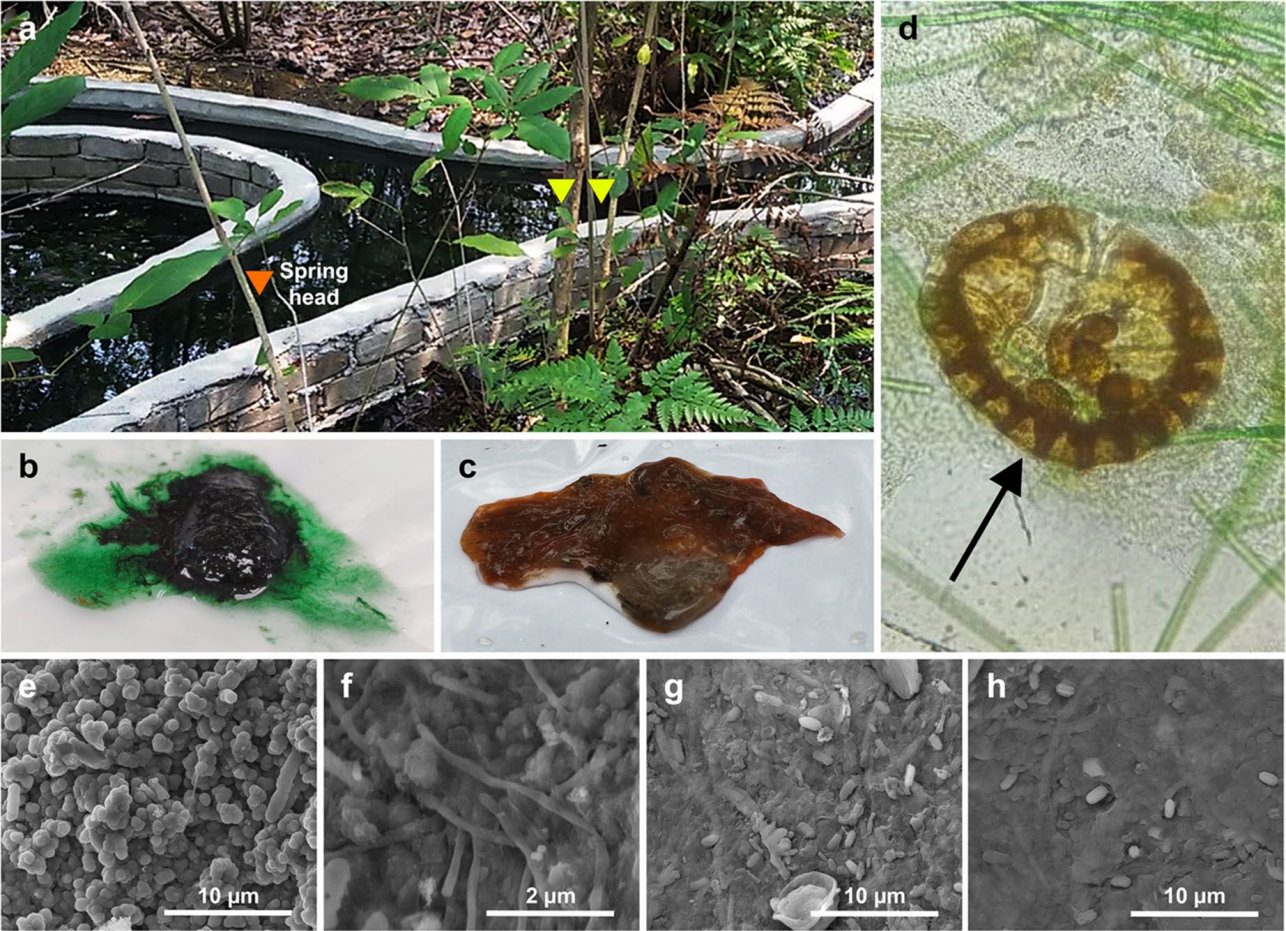
SKY hot spring. (**a**) SKY hot spring. Triangles indicate sampling sites. (**b**) Green microbial mat. (**c**) Brown microbial mat. (**d**) Light microscope (×400 magnification) of a green microbial mat. The sample contained filamentous microorganism and an unknown cell, putatively a eukaryote (indicated by an arrow). (**e**,**f**) Scanning electron microscope (SEM) images of green microbial mat. (**g**,**h**) SEM images of brown microbial mat.

The SKY hot spring is in the Malaysian woodlands. Three field trips (designated as 1, 2, and 3 hereafter) were conducted in Nov 2019, Feb 2020, and Aug 2020. The spring head is somewhere near the fork of the Y-shaped human-made feature (Fig. 1). Sediment at the spring head contained plant flakes or debris, fibre, brownish-reddish gelatinous microbial mat, pale tan biofilm, and occasionally some filamentous grey-whitish biofilm. Except for the region within ~ 0.5 m around the spring head, the rest of the hot spring is covered by a thin (< 5 mm) greenish microbial mat above a submerged plant litter bed^29^. We collected an emerald- green microbial mat at a location approximately three meters away from the spring head. The green (G) microbial mat and brown (B) samples were labelled as G1, G2, G3, or B1, B2, B3, respectively, to indicate which trip the samples were collected. Two separated samples and amplicon sequencing were performed for the first trip (Nov 2019), and the data were respectively designated as G1a, G1b, B1a, and B1b. The temperature during the samplings was 58–64 °C and 71–74 °C, respectively, for water adjacent to green and brown mats. The physicochemical parameters for the water are summarized in Table S1.

### Prokaryotic diversity in green and brown microbial mat using amplicon sequencing

**Figure 2:**
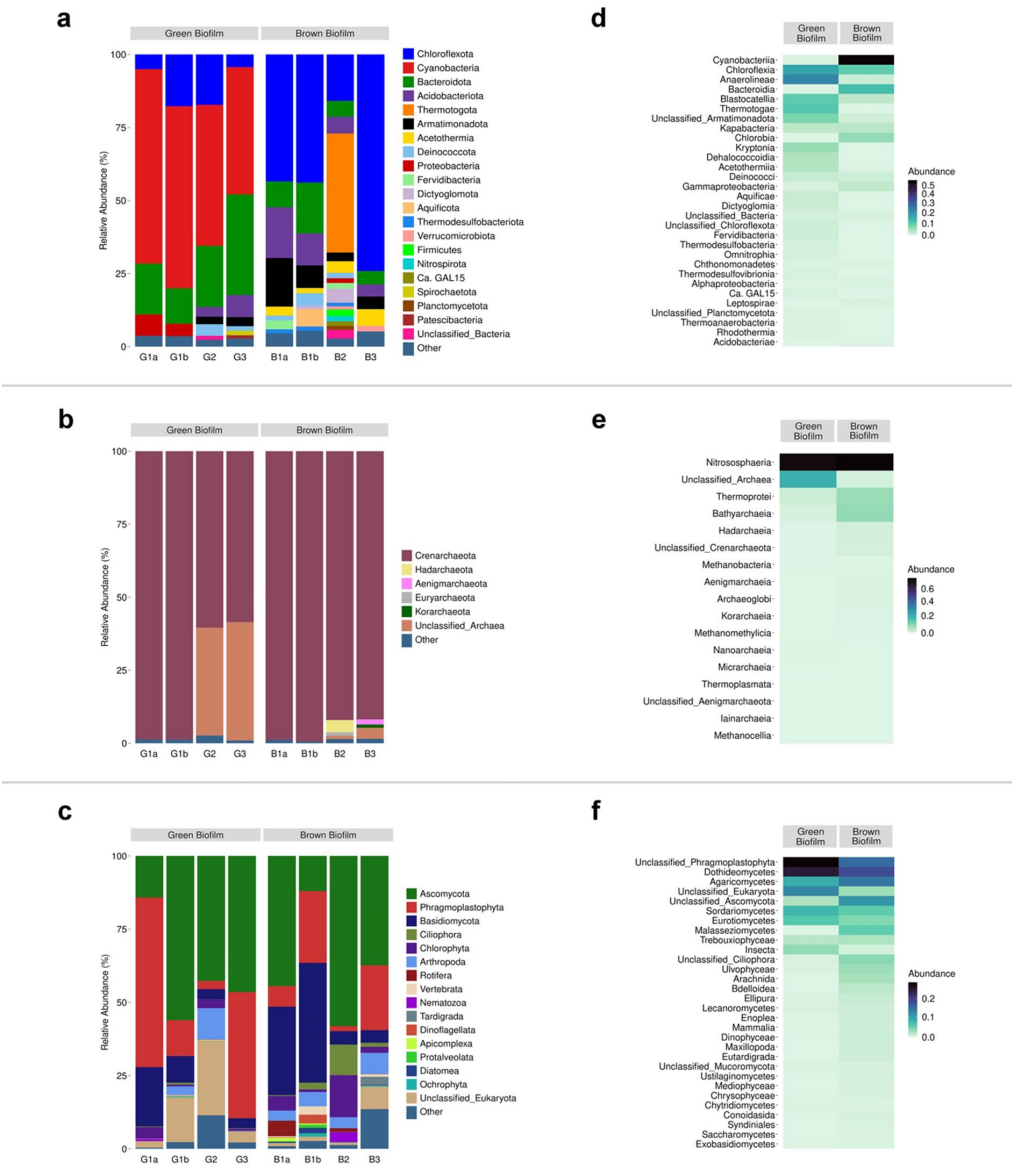
Taxonomic distribution at the phylum level of (**a**) Bacteria. (**b**) Archaea. (**c**) Eukaryotes. (**d**–**f**) Heat map showing the average relative abundance of class-taxonomy for green and brown mats, respectively for bacteria, archaea, and eukaryotes.

The overall bacteria metataxonomy identified using V3-V4 bacteria primers is summarized in Fig. 2a. Filamentous Cyanobacteria and Chloroflexota were the main phyla, and Cyanobacteria constituted 62–66% ASVs (amplicon sequence variants) in sample G1, 48% in G2, and 44% in G3. At least 12 various Cyanobacteria ASVs were annotated. Another major phylum was Bacteroidota (12–34% of total ASVs), with class Bacteroidia being relatively higher in G1, while Chlorobia and Kapabacteria were the dominant classes for G2 and G3.

Twenty-six bacteria phyla (> 1% of total ASVs) were identified in all brown microbial mats. Phyla Acidobacteriota, Armatimonadota, and Bacteroidota contributed 4–17% of the brown mat community (Fig. 2a). Generally, the brown-mats were dominated by phylum Chloroflexota (43% in B1, 16% in B2, and 74% in B3), and consisted of Anaerolineae, Chloroflexia, and Dehalococcoidia classes. Additionally, hyperthermophilic Thermotogota (with two ASVs of *Fervidobacterium* spp.) was another dominant phylum that constituted 41% of the total bacteria population in sample B2. However, *Fervidobacterium* appeared to be less than 0.2% in B1 and B3 samples. The collected brown samples were highly heterogeneous but abundant with hyperthermophiles. On average, the Shannon index for green- and brown-mat was 3.7 and 4.0, respectively.

The overview of archaea, after removing bacteria ASVs, is summarized in Fig. 2b. Phylum Crenarchaeota dominated brown mats. The average Shannon index for green and brown samples was 2.0 and 3.8, respectively. Green mats consisted of a relatively lower percentage of archaea domain to that of brown samples.

### Eukaryotic diversity in green and brown microbial mat using amplicon sequencing

Instead of ITS, 18S rRNA marker was used as it may provide a better comprehensive view on the total eukaryotes. Fungi ASVs monopolized the reads. The primary fungi phyla in the green microbial mat were Ascomycota, dominated by Dothideomycetes, Eurotiomycetes, and Sordariomycetes classes (Fig. 2c,f). Most fungi ASVs phyla dominated brown samples were Ascomycota and Basidiomycota. The dominant (> 5%) classes were Dothideomycetes, Eurotiomycetes, Lecanoromycetes, Sordariomycetes, Agaricomycetes, and Malasseziomycetes.

There were two dominant algae in the green mat, including the solitary green microalgae *Heveochlorella hainangensis* (phylum Chlorophyta) and unicellular green photosynthetic *Trebouxia usneae* (class Trebouxiophyceae). The main detected algae phyla in the brown microbial mat were Apicomplexa, Ciliophora, Diatomea, Dinoflagellata, and Protalveolata. In SKY hot spring green microbial mat, we noticed ASVs of Amoebozoa, Ciliophora, Protalveolata, and Ochrophyta.

### Taxonomy of shotgun assembled contigs

**Figure 3:**
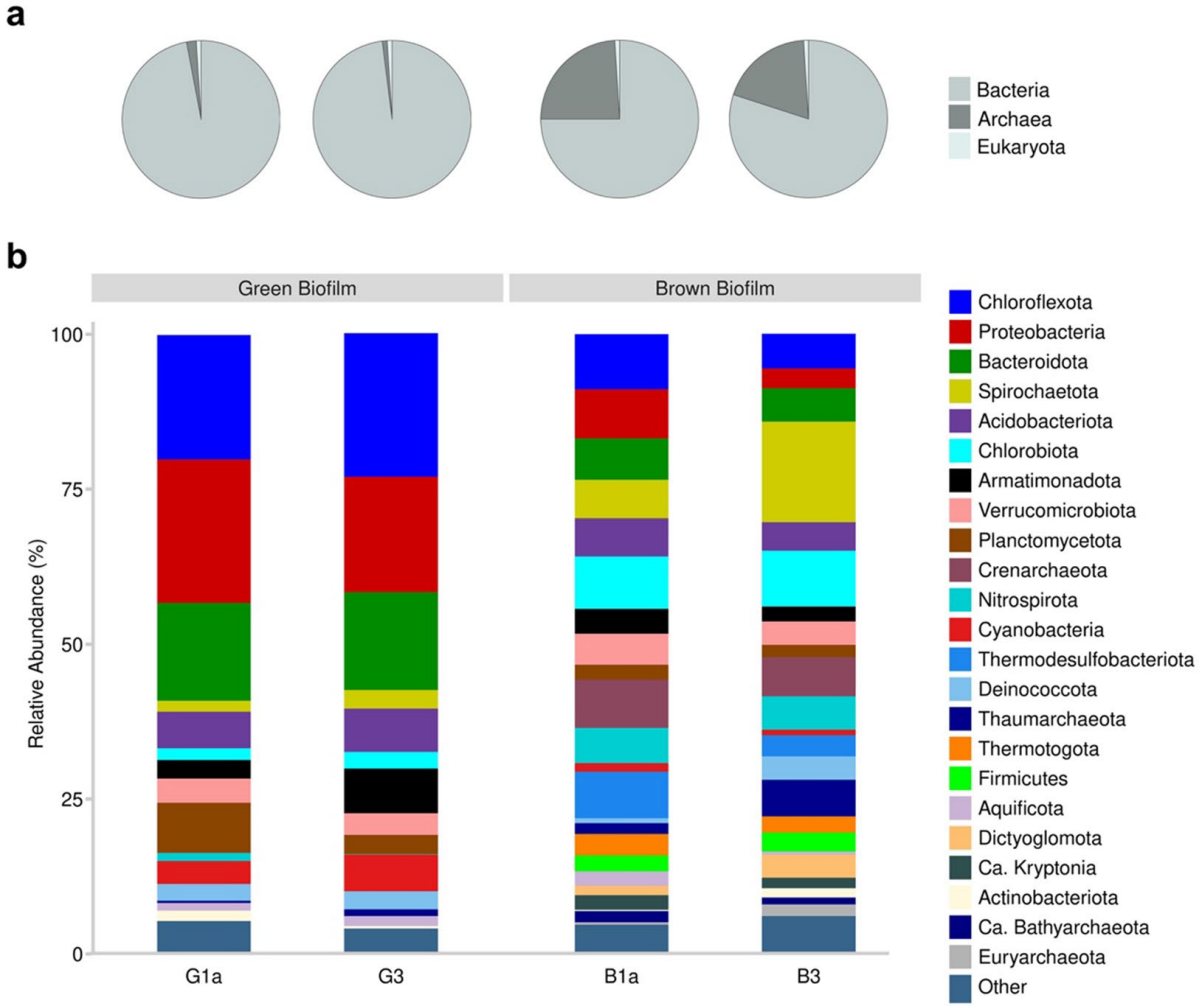
Overview of shotgun data. (**a**) Percentage of assembled ORFs assigned to bacteria, archaea, and eukaryotic proteins. (**b**) Main prokaryotes at phyla level.

Samples G1a, G3, B1a, and B3 have individually undergone shotgun metagenomic sequencing and reads were assembled using metaSPAdes into respectively 254,331, 107,238, 81,154 and 125,514 contigs that were larger than 500 bp. After filtering the unassigned taxa, approximately 98% of green mat contigs belonged to bacteria, while the remaining were archaea or eukaryotes. The average contigs for bacteria, archaea, and eukaryote in the brown mat were 77%, 22% and 1%, respectively (Fig. 3).

### Carbohydrates-degradation enzymes detected in shotgun assembled contigs

**Figure 4:**
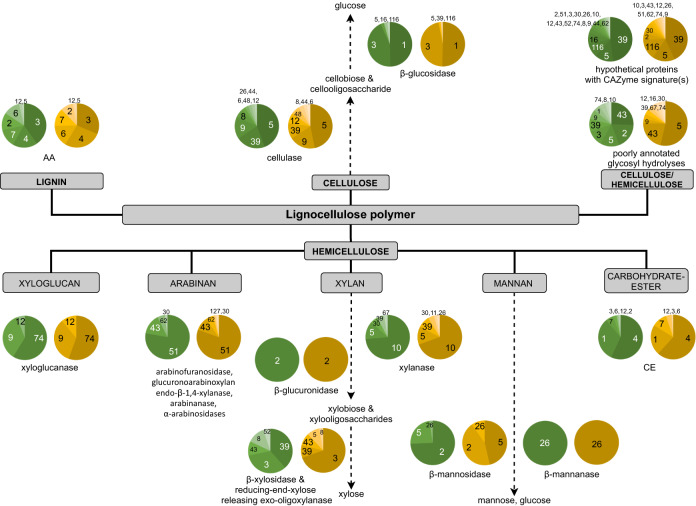
Putative lignocellulosic degradation pathway. Chart with green and beige shades refer to green- and brown mat, respectively. Numbers in each pie chart refer to families of AA, CE, or GH (i.e., GH5 and GH10 is indicated by 5 and 10, respectively).

One primary purpose of current work is to data-mine CAZymes, especially lignocellulosic enzymes, that may aid in the decomposition of plant litter in SKY hot spring. Open reading frames (ORFs) identified in the MetaSpades-assembled contigs were translated to protein sequences prior annotated against the CAZy database using dbCAN^35^. The putative enzymatic function of each CAZymes sequences was also predicted by sequence homology against NCBI non-reductant (nr), Uniprot reviewed protein database (Swiss-Prot), and InterProScan. The overall number of detected protein sequences are summarized in Table S2 at various levels of subject coverage and sequence identity. The total number of genes assigned to CAZymes observed in G1, G3, B1, and B3 were 15,668, 9859, 6719, and 10,348, respectively. In general, GH and glycosyl transferase (GT) family’s enzymes was the major CAZymes in green and brown samples. The numbers presented for GH in Table S2 incorporated cellulolytic- and non-cellulolytic glycosyl hydrolases. Table 1 summarizes the numbers and families of enzymes GH, CE and AA presumably performing lignocellulosic hydrolysis and bioconversion. Collectively, CAZymes involved in the putative lignocellulosic degradation in the green and brown microbial mat are illustrated in Fig. 4. The annotated glycoside hydrolases sequences include cellulase, endoglucanase, cellodextrinase, and β-glucosidase that putatively act on β-d-Glu-(1 → 4) linkages in cellulose, cellooligosaccharide or cellobiose. Primary enzymes acting on the β-d-Xyl-(1 → 4) of hemicellulase (xylan, oligoxylobiose, and xylobiose) are α-glucuronidase, xylanase, β-xylosidase, and reducing-end-xylose releasing exo-oligoxylanase. Arabinan active enzymes, for instance, α-l-arabinofuranosidase, glucuronoarabinoxylan endo-β-1,4-xylanase, exo-α-1,5-l-arabinanase, endo-α-1,5-l-arabinanase, and bifunctional β-xylosidase/α-arabinosidase, putatively target the α-d-(1 → 5)-Araf, α-d-(1 → 3)-Araf, or other linkages. Other detected protein sequences, xyloglucanase, β-mannosidase, β-mannanase, β-glucuronidase, β-N-acetylhexosaminidase, presumably conduct hydrolytic reactions at the other part of hemicellulase.

**Table 1:** Number of sequences associated with GH, CE, and AA families that were putatively associated with lignocellulosic hydrolysis and bioconversion.

Glycoside hydrolase (GH)	G1^a^	G3^a^	B1^a^	B3^a^	Green mat (G1 + G3)^b^	Brown mat (B1 + B3)^b^
GH1	72	87	58	87	17	12
GH2	83	62	55	78	0	0
GH3	143	87	92	137	9	5
GH5	92	63	67	121	6	9
GH6	3	0	2	2	0	0
GH8	9	3	2	5	1	0
GH9	19	8	14	20	1	1
GH10	25	17	22	41	2	0
GH11	1	0	1	3	0	0
GH12	13	6	5	14	0	0
GH16	43	20	14	22	1	0
GH26	6	2	4	9	0	0
GH30	9	7	6	12	0	0
GH39	155	90	47	90	0	0
GH43	24	16	7	22	4	1
GH44	3	1	3	4	0	0
GH48	0	0	3	6	0	0
GH51	36	33	41	60	6	3
GH52	3	2	3	4	0	0
GH62	2	1	3	5	0	0
GH67	1	0	2	5	0	0
GH74	16	7	3	8	0	0
GH116	26	22	24	39	2	1
GH141	19	15	19	46	0	0
**Carbohydrate esterase (CE)**						
CE1	287	169	63	125	13	6
CE2	2	0	0	0	0	0
CE3	16	11	2	3	0	0
CE4	197	105	74	111	5	9
CE6	9	3	2	3	0	0
CE7	1	27	23	36	5	2
CE12	8	4	1	6	0	0
CE16	1	0	0	0	0	0
**Auxiliary activity (AA)**						
AA1	114	103	62	86	0	0
AA2	15	10	7	5	5	2
AA3	75	41	20	26	7	4
AA4	94	53	92	97	1	2
AA5	2	1	0	1	0	0
AA6	37	20	48	74	2	6
AA7	46	33	31	35	6	0
AA10	0	0	1	1	0	0
AA12	9	4	5	7	1	2

^a^Blastp subject coverage ≥ 50%, identity ≥ 50%, and amino acid length ≥ 100.

^b^Non-redundant sequences with Blastp subject coverage ≥ 90%, identity ≥ 90%, and amino acid length ≥ 100.

Subsequently, we mined putative protein sequences that exhibited ≥ 90% subject coverage and ≥ 90% protein sequence identity, that were affiliated with lignocellulosic related GH families 1, 3, 5, 8, 9, 10, 16, 43, 51, 74, and 116 (Table 1). The annotated sequences include β-glucosidase, endoglucanase, cellobiohydrolase (exo-glucanase), cellulase, xylanase, α-N-arabinofuranosidase, α-l-arabinofuranosidase, glucan 1,4-α-glucosidase, β-mannosidase, β-xylosidase, β-glucuronidase, glycoside hydrolases and others. Forty-nine unique sequences (green mat) were associated with the following phyla: Armatimonadota, Chloroflexota, Deinococcota, *Ca.* Thermoflexus japonica, Cyanobacteria, Acidobacteriota and two unculturable bacteria. Thirty-two unique sequences encoding lignocellulosic-acting CAZymes that were ≥ 90% subject coverage and ≥ 90% protein sequence identify were spotted in the brown microbial mat. In addition, genes assigned to GH, CE, and AA were also noticed in different extensions from the following: Acidobacteriota, Bacteroidota/Chlorobiota group, Cyanobacteria, Firmicutes, Nitrospirota, Planctomycetota, Proteobacteria, Thermodesulfobacteriota, *Ca.* Thermoflexus japonica, Verrucomicrobiota, unclassified bacteria/unculturable, and several candidates from the division of Bathyarchaeota and Kryptonia. Among the listed GH enzyme sequences, only a few homologues have been characterized biochemically. For instance, protein sequence G1_212042 is a homologue to a characterized β-glucosidase AmBGL17 cloned from a soil metagenomic fosmid library^36^.

With ≥ 90% subject coverage and ≥ 90% protein sequence identity threshold, the total non-redundant CE sequences (family CE1, 4, 7, and 9) annotated in the green and brown mat datasets were 23 and 21, respectively. These CE families comprised esterase, polysaccharide deacetylase, acetylxylan esterase, and N-acetylglucosamine-6-phosphate deacetylase, often assist in xylan breakdown. With the same threshold, AA (family AA2, 3, 4, 6, 7, and 12) sequences present in the green and brown mat dataset were 22 and 16, respectively. The identified proteins were catalase-peroxidase, oxidoreductase, glycolate oxidase and sorbosone dehydrogenase.

### Cellulosic degradation enzymes with high subject coverage but low sequence identity

**Figure 5:**
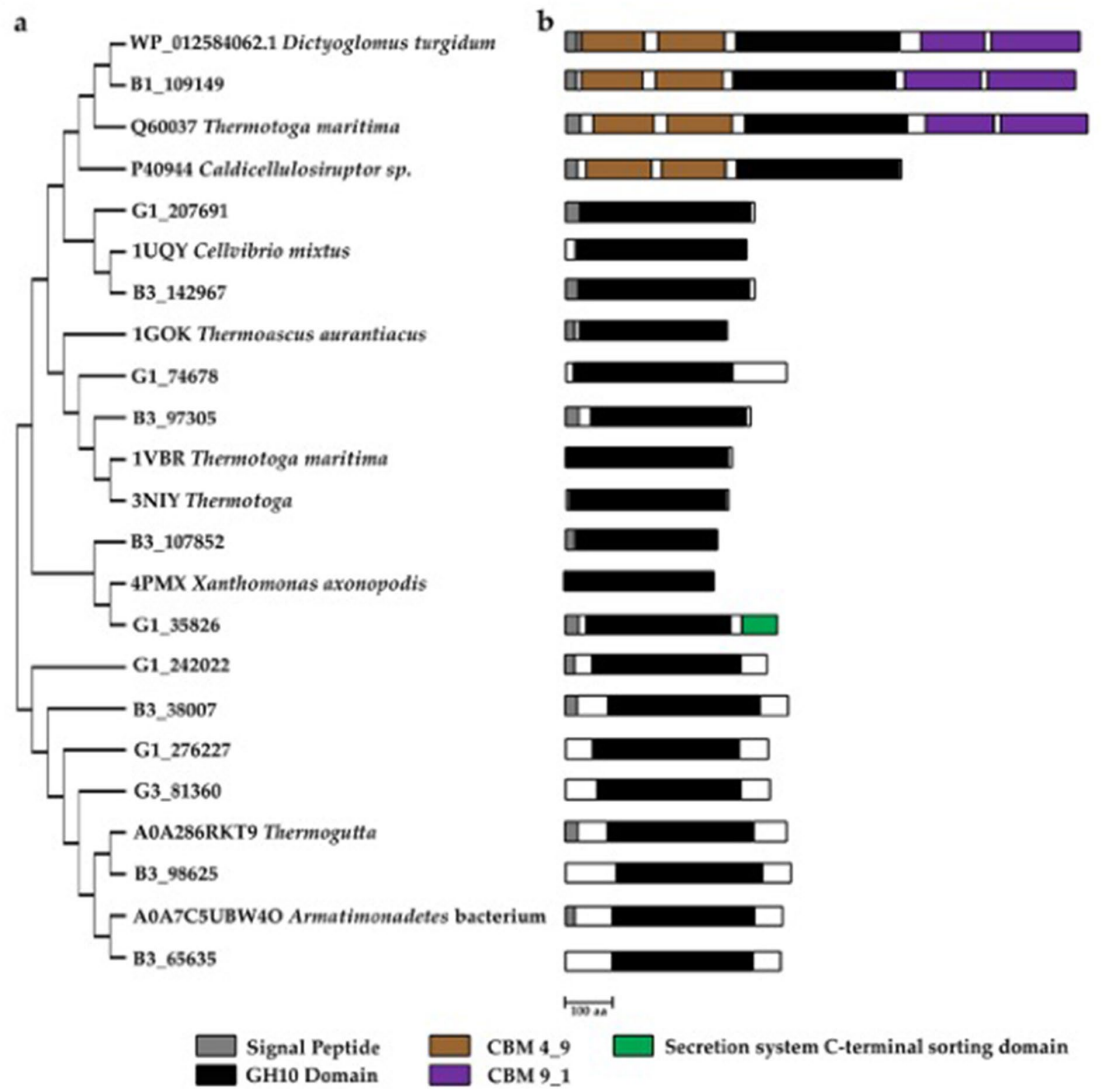
(**a**) Dendogram of xylanases. (**b**) Domains in xylanases.

**Figure 6:**
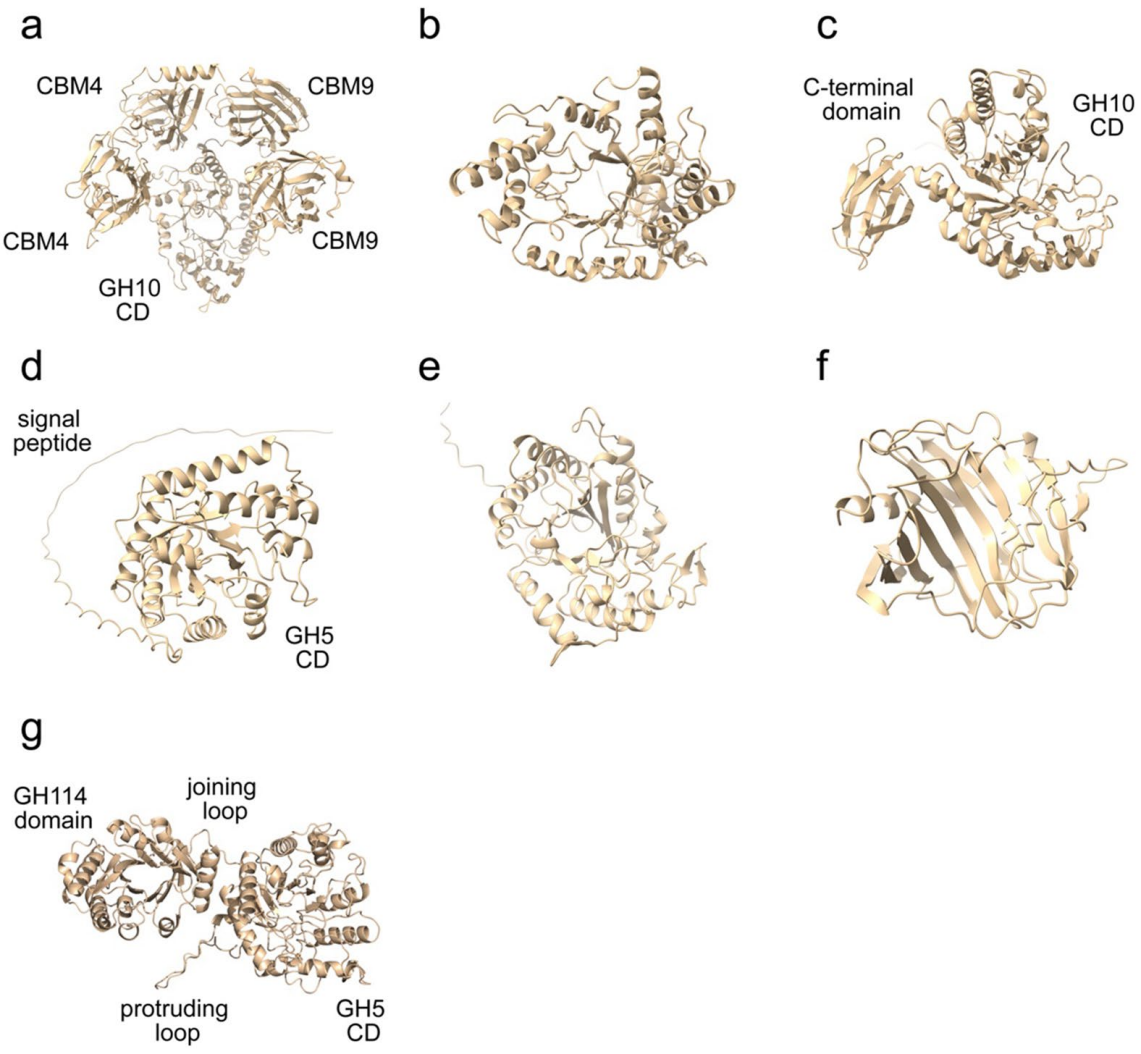
Predicted protein structures of selected CAZYmes using AlphaFold, an artificial intelligence program developed by Google’s Deepmind. (**a**) putative xylanase B1_109149, (**b**) xylanase G1_242022, (**c**) xylanase G1_35826, (**d**) cellulase G3_96404, (**e**) cellulase B3_136450, (**f**) cellulase B3_230401, and (**g**) cellulase B3_106662.

**Figure 7:**
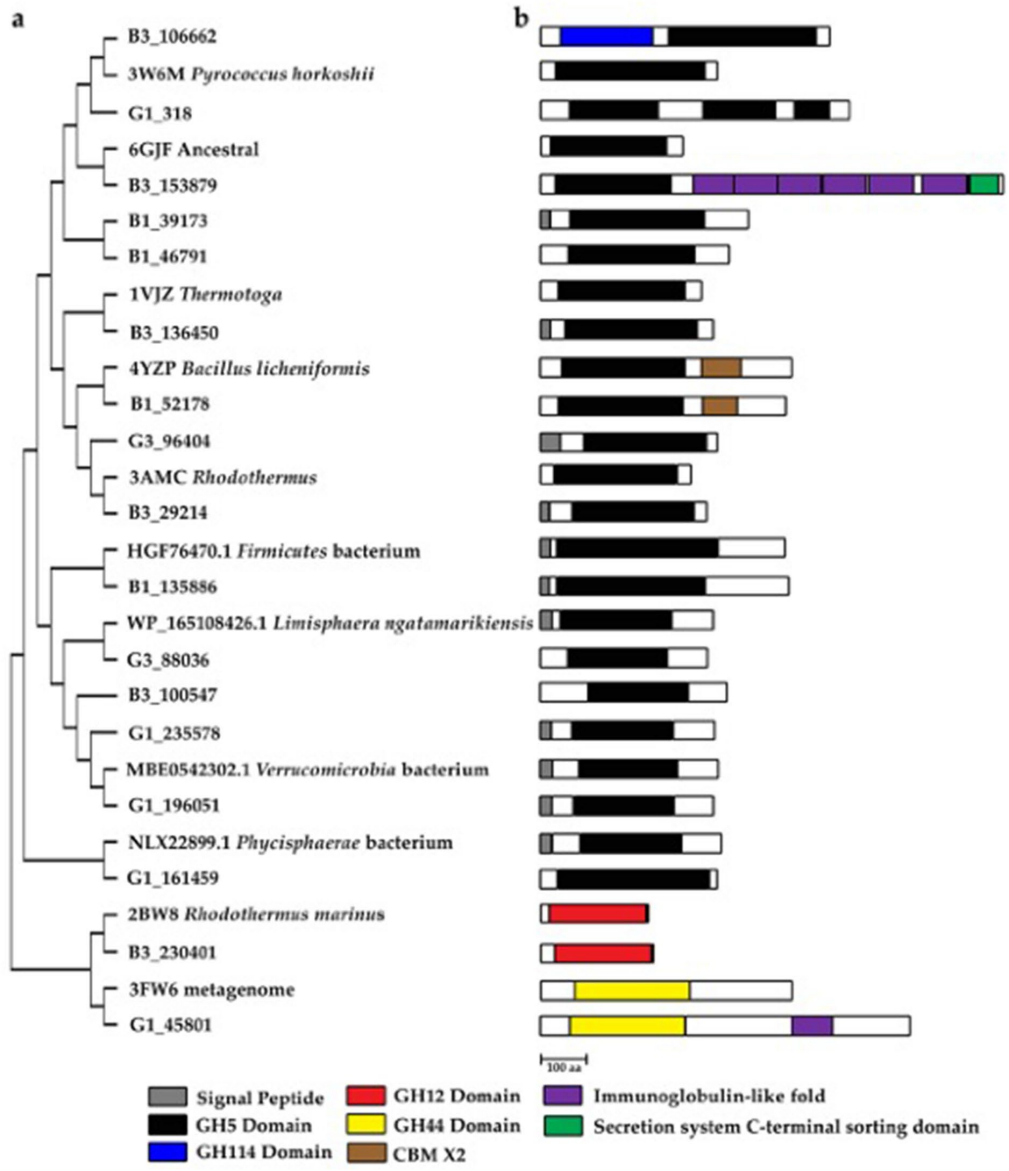
(**a**) Dendogram of GH5 cellulases. (**b**) Domains in cellulases.

In an earlier report^3^, the authors suggested that the sequences obtained from a metagenome assembly are considered novel if the primary sequence has ≥ 90% subject coverage and 50–70% identity to the deposited protein sequences. Using this as our threshold, we cataloged 116 and 91 non-redundant GH sequences in the green and brown samples, respectively. Approximately 80% of these sequences were related to families GH1, 3, 5, 10, and 51, while the remaining were from GH6, 8, 9, 12, 16, 26, 30, 43, 44, 48, or 116. We then selected some novel β-xylanase and cellulase and were compared with sequences obtained from various databases. Figures 5, 6, 7 show their positions in dendograms, domain architectures, and putative protein structure predicted using AlpaFold v.2. The protein fasta sequences were provided in supplementary material Fig. S1.

## Discussion

Phyla Bdellovibrionota, Fusobacteriota, and Myxococcota were present in the green microbial mat but in negligible quantities in the brown mat. The unique phyla detected in the brown mat, but not in the green microbial mat, included Caldatribacteriota, Thermodesulfobacteriota, Dictyoglomota, Elusimicrobiota, Thermotogota, Candidatus Calescamantes, Fervidibacteria, Hydrothermae, GAL15 and TA06. The Candidatus Caldatribacterium (phyla Caldatribacteriota), earlier named OP9 was also detected in this work. Using single-cell and metagenome sequencing, data elucidated that *Ca.* Caldatribacterium conducts anaerobic sugar fermentation and exhibited diverse glycosyl hydrolases, including endoglucanase^37^.

Cyanobacteria and Chloroflexota were the main identified phyla in the green microbial mat. Because the hot spring is almost stagnant, undisturbed, and the water surface temperature (< 64 °C) is below the maximum threshold of the bacteria photosynthesis process^38^, together these factors favor the growth of the microorganisms. Chloroflexota *Thermoflexus hugenholtzii*^39^ (opt. growth temp. [OGT] 72–75 °C) constituted 31% of B1 microbiota (Fig. 2). The complete genome of *T. hugenholtzii* JAD2^T^ and several associated metagenome-assembled genomes are available^40^, and they harbored multiple cellulosic degrading enzymes. When we extracted the DNA materials from sample B3, approximately half of the working materials was reddish-brown jelly-type microbial mat while the remaining were heterogeneous materials. 63% of the total ASVs in the B3 mat were dominated a taxon related to *Roseiflexus*, another Chloroflexota member. At the time of writing, *Roseiflexus castenholzii* HLO8^T^ (DSM 13941) is the only described type strain. Bacterium HLO8^T^, a photosynthetic strain, formed a reddish-brown microbial mat in a Japanese hot spring^41^. We anticipate that the Chloroflexota associated taxon that formed reddish-brown jelly-type microbial mat in the spring head of SKY hot spring (71–74 °C) is different from strain HLO8^T^ (OGT 55 °C) as the latter could not thrive at a higher temperature^41^.

*Fervidobacterium*, under the Thermotogota phylum, was a major genus in sample B2. *Fervidobacterium* species, for instance, *F. islandicum* and *F. changbaicum*, exhibit a broad range of CAZymes. The percentage of *Fervidobacterium* in hot spring microbiota would be increased if the water was enriched with switchgrass inoculum^32^. Some *Firmicutes* ASVs were detected in the brown mats. Firmicutes’ members, i.e., *Geobacillus* and other Firmicutes bacilli thermophiles, may dominate cellulose-degrading consortium in a heated lab setup^42^. *Caldicellulosiruptor thermophilum*, another member of *Firmicutes*, has been targeted as one potential thermophile for consolidated bioprocessing of lignocellulose^2^. We detected *Caldicellulosiruptor* and other *Firmicutes* in relatively small quantities in SKY hot spring mats.

Crenarchaeota was the dominated Archaea phylum in brown mats, with Nitrososphaeria being the main class and consisted of *Ca.* Caldiarchaeum and *Ca.* Nitrosocaldus (Fig. 2). The knowledge on these candidates is very limited^43–45^. The remaining classes in brown samples were Bathyarchaeia and Thermoprotei. ASVs stated above were also present in green microbial mats with the exception of Nitrosocaldales which was the main order in green biofilm datasets but existed in relatively smaller quantity in brown mats.

The study of eukaryotes is scarce for hot springs around the globe and is often neglected compared to prokaryotes. Oliverio et al. examined the presence of protists (microbial eukaryotes) in 160 New Zealand geothermal springs and suggested that the main protists possibly thrived in elevated temperatures are Amoebozoa, Archaeplastida, Alveolata, Excavata, Rhizaria, and Stramenopiles^20^. In SKY hot spring green microbial mat, we noticed ASVs of Amoebozoa, Ciliophora (protozoa algae that feed bacteria), Protalveolata, and Ochrophyta. The Amoebozoa *Echinamoeba thermarum* was a common thermophilic protist in New Zealand geothermal springs^20^. *E. thermarum* was positively identified in the green mat but absent in the brown mat. We spotted other Amoebozoa, for instance, taxa from order Euamoebida and Leptomyxida. Additionally, thermophilic protist Protalveolata (mainly from class Syndiniales) was approximately 1% of total eukaryotes ASVs in the B1b sample. Thermophilic protist Ochrophyta (particularly class Chrysophyceae) was detected in very small quantity in brown samples.

18S rRNA metataxonomic sample datasets elucidated that thermophilic fungi were present only as the minority (< 1% ASVs). That included *Chaetomium* (T_max_ 61 °C), *Paecilomyces* (55 °C), *Chrysosporium* (60 °C), *Trichothecium* (57 °C), *Paecilomyces* (55 °C), *Torula* (58 °C), *Talaromyces* (50 °C), *Paecilomyces* (50 °C), *Geosmithia* (55 °C), and *Thermomyces* (60 °C)^46^. We were doubtful if all detected fungi ASVs could grow pleasantly in SKY hot spring. Also, the water level was low during the first field trip, and the green mat was not submerged completely, water temperature was 58 °C, as measured ~ 5 cm below the G1 green mat. The actual temperature was expected to be lower in the floating green microbial mat; therefore, certain thermophilic fungi or some heat-tolerant mesophilic fungi may survive. However, none of the currently known thermophilic fungi can develop beyond 70 °C; therefore, detected taxa are not likely to survive without sporulation at the SKY brown mat at the spring head. Moreover, it is suspected that most of the detected mesophilic fungi ASVs were originated from fallen plant litter, and we expect them to be in their dormant form. For the first time, microscopic water bear Tardigrada (*Macrobiotus hufelandi*) was detected in a Malaysian hot spring. This small eight-legged animal feed on microorganisms, decomposed leaf, and survive in extreme temperatures. We also detected high background of plant phyla Phragmoplastophyta that likely originated from plant debris. Other background ASVs included flies, grasshoppers, and fringed winged insects, mites, and ticks. We confirmed that eukaryotes were the minority group in the SKY hot spring microbial mats by putting together the shotgun and amplicon data. We think that 18S rRNA primers excessively amplified the background of plant residuals or chromosomal fragments from the dead organisms, spores, inactivated eggs or larva in particular mesophilic fungi or insects. Collectively, we concluded that relatively high background noise was observed using 18S rRNA primer set.

We performed shotgun metagenomic sequencing using two green- and two brown microbial mats. Negligible amounts (~ 0.1%) of virus reads were detected in all the mats, and it is quite common to see a trace quantities of viruses in hot springs^47^. Judged using contigs generated from shotgun sequencing data, a greater percentage of archaea present in the brown mat was probably related to a higher temperature at the spring head (Fig. 3). We also think that temperature is the main abiotic factor that differentiate the microbial profile in green- and brown mats. In addition, data elucidated that bacteria are the main microbiota in green and brown mats, and they are the primary plant-biomass degraders and consumers in SKY hot spring. This observation was also noticed earlier in a separate report^16^. Despite lower abundance and diversity, archaea and some candidate taxa may exhibit some functional role on lignocellulosic decomposition in SKY hot spring. Several sequences of CAZymes from GH families (i.e., GH1, 3, 5, 10, etc.) were identified from Candidatus Bathyarchaeota, Candidatus Brockarchaeota, Thaumarchaeota, Nitrososphaeria archaeon, and Thermoprotei archaeon.

On average, more than 10,000 CAZymes ORFs were found in each type of microbial mat (Table S2). The annotated glycoside hydrolases sequences included cellulases, hemicellulases, CEs, GTs, AAs, and enzymes acting on carbohydrates such as starch. More cellulase and hemicellulase sequences were identified in SKY hot spring than the counterpart numbers detected in an Indian hot spring metagenomic study using water–sediment samples lacking in-situ plant litters^3^. Besides, the metataxonomic described in this current study differed from Deulajhari hot springs and Obsidian Pool that contained Pandanus leaf litters and heat-tolerant plant *Juncus tweedyi*, respectively^16,28^. The microbial and enzyme diversity in SKY hot springs are far more complex than other heated in-situ or ex-situ studies supplemented with insoluble cellulosic biomass^31–33,42^.

Using the threshold of ≥ 90% subject coverage and ≥ 90% protein sequence identity, the three main phyla essential for biomass degradation were Chloroflexota, Armatimonadota, and Deinococcota. The primary contributors (phylum Chloroflexota) were taxa related to *Roseiflexus castenholzii*, candidatus taxa, and a few unclassified *Chloroflexota* bacteria. Other important Chloroflexota taxa for high-temperature lignocellulosic degraders included *Thermomicrobium roseum*, *Anaerolineae*, *Ardenticatenia*, *Caldilinea aerophile*, *Ca.* Thermofonsia Clade 1, *Chloroflexus islandicus*, *Thermoflexales*, and *Thermoflexia* bacterium. According to the online CAZy genome databases, members of the Chloroflexota phylum exhibited a broad range of GH enzymes. For instance, the anaerobe photoheterotrophic thermophilic *R. castenholzii* DSM 13941 (NCBI genome accession CP000804.1) has 22 GH families and 3 CE families that accounted for 60 different protein sequences^41^.

Armatimonadota is another phylum spotted with multiple sequences from GH1, 5, 10, 43, and 51, CE7, and AA12 (threshold: ≥ 90% subject coverage and ≥ 90% protein sequence identity). Using the metataxonomy dataset, at least four Armatimonadia ASVs were detected in SKY hot spring mats, and an ASV was closely related to class Chthonomonadetes while the rest were unresolved at the lower taxonomy level. This phylum, earlier designated as candidate division OP10, was initially found in Yellowstone National Park Obsidian Pool^48^. Currently, *Chthonomonas calidirosea* T49^T^ is the only thermophilic type strain^48^. The complete genome of strain T49^T^ harbored 64 glycosyl hydrolases and eight carbohydrate esterases. To the best of our knowledge, the characteristics of these enzymes are still undescribed.

Deinococcota is the third-largest phylum with 28 sequences with ≥ 90% subject coverage and identity to the CAZy database. These putative sequences have high identity to counterpart proteins from *Calidithermus*, *Thermus*, and *Meiothermus* species. The draft genome of *Calidithermus timidus* DSM17022 indicated that the bacterium harbor seven AA, 38 GH, 16 CE sequences^49^. So far, only a GH57-glycogen branching enzyme^50^ and GH13 amylosucrase^51^ from this bacterium have been analysed in detail. *Meiothermus* spp. may help break down plant litter in SKY because a representative, *M. taiwanensis* WR-220 (PRJNA205607), had enzymes such as xylanase, β-xylosidase, endoglucanase, and polysaccharide deacetylase. More than a dozen *Thermus* spp. have completely curated genomes in the CAZyme genome database. For an example, the genome of *Thermus thermophilus* (http://www.cazy.org/b12268.html) encoded sequence of 15 types of enzymes particularly from families GH1, 13, 23, 36, 57, 63 and 77; however, the essential enzyme for lignocellulose hydrolysis is missing. Therefore, *Thermus* spp. are sugar consumers in the SKY community.

We are interested in mining novel CAZymes from the shotgun contigs (Fig. S1). A protein sequence may be considered novel if the primary sequence has ≥ 90% subject coverage and 50–70% identity to the deposited protein sequences. We spotted a 1036-residues β-xylanase B1_109149 with approximately 60% identity with an endo-1,4-β-xylanase (WP_012584062.1) from thermophilic *Dictyoglomus turgidum*. Both protein sequences formed a cluster with β-xylanase from *Thermotoga maritima* (Q60037) (Fig. 5). All three sequences contained a signal peptide, two N-terminal β-sandwich fold CBM4_9, followed by a TIM barrel GH10-catalytic domain consisting of four conserved motifs and with two β-sandwich CBM9_1 at the C-terminal. The putative protein structure of β-xylanase G1_109149 was predicted using AlpaFold v.2^52^ and is shown in Fig. 6a. Additionally, another twelve GH10 family putative novel β-xylanase sequences were present in the dataset. These proteins sequences were probably related to phyla Armatimonadota, Bacteroidota/Chlorobiota, *Ca.* Bipolaricaulota, *Ca.* Solibacter, Ignavibacteriota, Planctomycetota, or Verrucomicrobiota (Fig. 5). The identified β-xylanase sequences have a single domain of the GH10_2 family where the four conserved motifs were located. The predicted structures of the selected xylanases are shown in Fig. 6b–d.

The primary sequence of β-xylanase G1_35826 is unique because it has a domain related to the secretion system C-terminal sorting domain and is absent in other counterparts displayed in Fig. 5. The other name for that domain is por-secretion system or the T9SS type IX secretion system. The putative protein structure of β-xylanase G1_35826 is displayed in Fig. 6c, and the C-terminal domain resembled a β-sandwich fold structure. There is little research exploring how annotated xylanase is incorporated with a T9SS. We observed such domain in xylanase XynRA2 from halo-thermophilic *Roseithermus* sp., and xylanase Xyn10A from *Rhodothermus marinus*, and xylanase Xyl2091 from *Melioribacter roseus*^53^. All these thermo-halophilic bacteria are from Bacteroidota/Chlorobiota group. Based on a recent review, certain microorganisms, especially those from Bacteroidota utilise the T9SS system for secreting proteins^54^.

Subsequently, we data-mined novel cellulase sequences in the SKY hot spring dataset. Fourteen unique sequences are putative cellulases, and each of the sequences contained a GH5 domain. Putative cellulase G3_96404 was 55% homologous to cellulase Cel5A of *T. maritima* (PDB 3AMC)^55^. 3AMC structure has a classic TIM barrel fold that resembles endoglucanase TM1752 (1VJZ) from *T. maritima*, endocellulase EGPh (3W6M) from *Pyrococcus horikoshii* and endoglucanase (6GJF) from a synthetic construct^56^. Cellulase EGPh has the optimum activity at 100 °C^57^. Putative cellulase B3_136450 (Figs. 6e and 7) has identical domain setups with the endo-β-1,4-glucanase BlCel5B sequence (4YZP) from *Bacillus licheniformis*^58^. BlCel5B was catalytically actived on CMC, β-glucan, lichenan, and xyloglucan. Protein BlCel5B has tri-modular structure with an N-terminal catalytic GH5 domain (18–320 amino acid stretch), an immunoglobulin-like module (345–428), and a C-terminal CBM46 (432–533)^58^. The immunoglobulin-like module with two β-sheets resembles an earlier known CBM_X2 that may be involved with cellulosome^59^.

Additionally, we annotated three cellulases that belong to non-GH5 groups (Fig. 7). B3_230401 (238 aa) was 50% homologous with the primary sequence of endoglucanase Cel12A (PDB 2BW8, 227 aa) from *Rhodothermus marinus*^60^. Both sequences have a single GH12 catalytic domain. Often, that domain is similar to the concanavalin-like glucanase domain superfamily that looks like a sandwich structure with 12–14 β-strands (Fig. 6f). Another putative cellulase G1_45801 sequence was 69% homologous to the sequence of a crystal structure CelM2 (3FW6), and Interproscan indicates that both sequences have identical domain arrangements. The gene of CelM2 was cloned from a metagenomic library^60^. GH44 domain (β-sandwich structure) was found in the N-terminal while a galactose-binding domain (TIM-like barrel structure) was present at the C-terminal, where the acid/base Glu 221 and nucleophilic Glu393 are located^61^. Enzyme CelM2 actively hydrolysed multiple substrates, including birchwood xylan, barley glucan and cellulosic CMC, respectively having β-1,3/4-glucan and β-1,4-glucan linkages^61^. The binding ability of multi-substrates is possibly related to the broad and deep pocket. Due to relatively close sequence identity, G1_45801 may exhibit a similar bifunctional glucanase-xylanase activity as CelM2. Lastly, putative cellulase B3_106662 (615 aa) was detected in a brown microbial dataset. The stretch 42–238 resembles a GH114-family domain having a typical aldolase-type TIM-barrel structure (Fig. 6g). The latter half of the sequence (residue 273–587) is the GH5 family domain, resembling the second TIM-barrel. A short loop (residue 247–254) joint both TIM-barrels. As shown in Fig. 6g, a long loop protruding from the GH5-TIM barrel that points towards the GH114-TIM barrel. We expect that the protruding loop has some structural role. So far, there are no closely related crystal structures to the sequence of B3_106662.

## Conclusion

More than a thousand hot springs metataxonomic data are deposited in databases. Only Obsidian Pool at Yellowstone Natural Park, Indian Deulajhari spring cluster, and SKY hot springs have reported plant biomass. Hot springs rich with lignocellulosic materials from natural ecosystem are reservoirs of metagenomic data to harness excellent carbohydrate degrading thermozymes. By incorporating amplicon and shotgun metagenomics, this study elucidated that green and brown microbial mat exhibit different microbial profiles, probably driven by temperature and other factors. Current data suggested that microbial profiles and enzymes involved in the lignocellulosic decomposition are more complex than initially thought and is more intricate than ex-situ heated experiments. Many of the taxa in SKY hot springs have not been cultivated. Green mat was rich with photosynthetic microorganisms i.e., Cyanobacteria and Chloroflexota. Brown microbial mat was highly heterogenous, and the microbial community was relatively more complex. Bacteria’s enzymes may play a more prominent role in high-temperature lignocellulosic degradation. Few archaea and Candidatus species, such as Candidatus Bathyarchaeota, Candidatus Brockarchaeota, Thaumarchaeota, Nitrososphaeria archaeon, and Thermoprotei archaeon, made a considerable contribution. Certain taxa, for instance, *Thermus*, were sugar-consumers. Both green and brown samples were rich with unexplored CAZymes. Few of the interesting putative lignocellulosic-glycosyl hydrolases and their counterpart homologous proteins were described in this report. This work expands our understanding on thermophilic lignocellulosic degradation and provides new enzyme targets for future development. Thermostable cellulases, xylanases, CE, and AAs could form a cocktail for biofuel industries.

## Methodology

### Sample handling, water analyses and DNA extraction

Y-shaped Sungai Klah (SKY) hot spring is located at 3°59′50.50′′N and 101°23′35.51′′E. The temperature was measured using a portable digital thermometer, and pH was measured at 25 °C. Water from the spring head was collected into Schott bottles for chemical analysis at Allied Chemists Laboratory Sdn. Bhd. During the first sampling (Nov 2019), the water level was relatively low with a depth of 25–30 cm, but the upper layer of the plant litter bed was sufficiently wet. Two samples of green microbial mats (labelled as G1a and G1b) (~ 20 cm apart) were collected from the upper layer of the bed that was approximately 3.5 m away from the spring head. Two samples of brown microbial mats (~ 10 cm apart at the spring head) were taken and labelled as B1a and B1b. During the second and third sampling trips (Feb 2020 and Aug 2020), the water level was higher (45–50 cm depth), and the collected green and brown microbial mat samples were designated as G2, G3, B2, and B3. All microbial mats were kept at cool during transportation and frozen at − 20 °C within 12 h.

### Total DNA extraction

Frozen samples were thawed at room temperature; and leaves and twigs were removed manually. Bulk genomes from green and brown microbial mat samples (G1a, G1b, G2, G3, and B1a, B1b, B2, B3) were extracted using the FastDNA Spin Kit for Soil (MP Biomedicals, Solon, USA). A 500 mg biofilm sample was resuspended in sodium phosphate and MT buffers as provided in the kit. The mixture was transferred into a Lysing Matrix E tube and mechanically lysed in a TissueLyser II (Qiagen, Hilden, Germany) set at 20 Hz for 5 × 3 min and subsequently centrifuged at 10,000×*g* for 5 min. The supernatant was then withdrawn and underwent the protocol suggested by the manufacturer. Extracted DNA was evaluated by a NanoDrop 1000 spectrophotometer, a Qubit 3.0 Fluorometer (Thermo Fisher Scientific, Waltham, USA), and a 1% (*w/v*) agarose gel electrophoresis.

### Amplicon sequencing and bioinformatic analyses

The extracted metagenomic DNA from the green samples (G1a, G1b, G2, and G3) and the brown samples (B1a, B1b, B2, B3) were used as templates. The following primers were used for PCR amplification: (a) bacterial 16S rRNA V3-V4 region (341F 5′-CCTAYGGGRBGCASCAG-3′ and 806R 5′-GGACTACNNGGGTATCTAAT-3′), (b) archaeal 16S rRNA V4 region (U519F 5′-CAGYMGCCRCGGKAAHACC-3′ and 806R 5′-GGACTACNSGGGTMTCTA AT-3′), and (c) eukaryotic 18S rRNA at V4 region (528F 5′-GCGGTAATTCCAGCTCCAA-3′ and 706R 5′-AATCCRAGAATTTCACCTCT-3′).

Amplicon sequencing was conducted using Illumina NovaSeq 6000 platform (Illumina, San Diego, USA) with paired-end 250 base pairs at NovogeneAIT Genomics (Singapore). A minimum sequencing depth of 100 K raw reads was reserved for every sample. The resulting raw paired-end reads were processed using the DADA2 plugin in QIIME 2 pipeline^62,63^. The process includes demultiplexing, quality-filtering, denoising, dereplication, and removal of chimeras, and clustering of the paired-end sequences. Taxonomy classification was carried out using SILVA SSU 138.1 database.

### Shotgun sequencing and bioinformatic analyses

DNA samples (G1a, B1a, G3, and B3) were fragmented by a Covaris sonicator (Covaris, Woburn, USA). Then, the fragmented DNA was used for dual-indexed, paired-end library construction following the Illumina DNA Prep kit protocol (Illumina, San Diego, USA). The constructed library samples were run in Qubit 3.0 Fluorometer and Agilent Bioanalyzer 2100 (Agilent Technologies, Palo Alto, USA). Whole metagenome shotgun sequencing was carried out in an Illumina NovaSeq 6000 with the running mode of PE 150 (paired-end 150 bp) conducted in NovogeneAIT Genomics (Singapore). A minimum of 20 Gb (equivalent to approximately 66.5 million paired end reads) output was reserved for each sample. The resulting raw paired-end reads were trimmed and filtered by SOAPnuke v2.1.6 software^64^. Clean paired-end reads were de novo assembled by metaSPAdes assembler v3.15.2^65^. Taxonomy classification on the assembled contigs was carried out by Kraken2 v2.1.2^66^. MetaQUAST v5.0.2 with default MetaGeneMark as gene predictor was used to find all the open reading frames (ORFs) from the assembled contigs^67^. Contigs < 500 bp were excluded. All ORFs were also subjected to Carbohydrate-Active enZYmes (CAZymes) annotation via run_dbcan v2.0.11 coupled with the latest CAZy database v07312020^35^. Sequences that were positive in at least one in program HMMER, Hotpep, and Diamond^35^ were shortlisted and further annotated by NCBI non-reductant (nr) and Uniprot reviewed protein database via Diamond v2.0.11.149^68^. Selected CAZymes were further analysed using InterProScan v5.52-86.0 for predicting domains, motifs, and others^69^. The motifs of *Thermotoga maritima* β-xylanase was used as the reference (IRGHTLVWHNQTP, VYAWDVVNEAVD, AKLFYNDYNTFE, and EKGLIDGIGMQCH). Protein structure prediction was performed by AlphaFold Colab v2.0^52^ using the default parameters and displayed using UCSF ChimeraX v1.2.5.

## Supplementary Information

Supplementary Information.

## Data Availability

The amplicon and shotgun sequencing data were deposited in the NCBI with BioProject number PRJNA761511 and BioSample accessions SAMN21353065–SAMN21353070.
