# Supplementary material for: Thermophiles and carbohydrate-active enzymes (CAZymes) in biofilm microbial consortia that decompose lignocellulosic plant litters at high temperatures
Source: Sci Rep. 2022 Feb 18;12:2850. doi: 10.1038/s41598-022-06943-9 (PMC8857248; doi:10.1038/s41598-022-06943-9)
Supplement: Supplementary file 1 — Supplementary Information. [file 41598_2022_6943_MOESM1_ESM.pdf]

Parameter	Method	Value	Unit
Temp.	Digital thermometer	58-74	°C
pH	APHA 4500 H^+ B	8.5	-
TOC	APHA 5310 C	0.8	mg/l
Total nitrogen as N	APHA 4500-N_{org} B & APHA 4500-NH_3 C	1.8	mg/l
Ammonical Nitrogen as N	APHA 4500-N_{org} B&C	<0.2	mg/l
Nitrate as NO_3^-	HACH nitrate test kit	<0.1	mg/l
Nitrite NO_2^-	APHA 4500 NO_2^- B	<0.1	mg/l
Phosphorus as P	USEPA 6010 B	0.1	mg/l
Sulfur as S	USEPA 6010 B	2.7	mg/l
Sulphate as SO_4^{2-}	APHA 4500 SO_4^{2-} E	5	mg/l
Sodium as Na	USEPA 6010 B	47	mg/l
Potassium as K	USEPA 6010 B	2.2	mg/l
Calcium as Ca	USEPA 6010 B	2.7	mg/l
Magnesium as Mg	USEPA 6010 B	0.2	mg/l
Bicarbonate as $CaCO_3$	APHA 2320 B	27	mg/l
Chloride as Cl^-	APHA 4500 Cl^- B	1	mg/l
Fluoride as F^-	APHA 4500 F^- D	1.3	mg/l
Copper as Cu	USEPA 6010 B	0.02	mg/l
Iron as Fe	USEPA 6010 B	0.02	mg/l

Table S1. Chemical analysis of water.

Samples	AA	CBM	CE	GH	GT	PL
All sequences classified by HMMER, Hotpep, or Diamond program						
G1	472	1902	1343	5471	8200	182
G3	307	1030	723	3392	5355	82
B1	275	618	398	2486	3496	61
B3	345	963	668	3939	5262	134
≥50% subject coverage, ≥50% protein sequence identity, ≥100 amino acid length						
G1	421	1208	1068	3940	5832	119
G3	283	731	611	2625	4081	64
B1	267	457	348	1988	2786	44
B3	321	706	568	3092	4184	111
≥90% subject coverage, ≥50% protein sequence identity, ≥100 amino acid length						
G1	328	779	718	2520	4044	73
G3	249	516	463	1992	3129	40
B1	237	334	250	1474	1972	32
B3	274	508	401	2262	3034	65
≥90% subject coverage, ≥50% protein sequence identity ≤70%, ≥100 amino acid length						
G1	63	339	282	841	1757	41
G3	40	162	121	467	1108	18
B1	30	77	36	263	674	5
B3	29	126	86	519	1003	27
≥90% subject coverage, ≥90% protein sequence identity, ≥100 amino acid length						
G1	72	78	85	370	471	2
G3	74	83	89	468	550	2
B1	73	49	52	308	292	-
B3	84	64	69	429	415	-

Table S2. Numbers CAZymes and carbohydrate-binding module at various subject-coverage and -identity. Auxiliary activities protein (AA), carbohydrate-binding module (CBM), carbohydrate esterase (CE), glycosyl hydrolase (GH), glycosyl transferase (GT), and polysaccharide lyases (PL). The numbers include the duplicated or homologues of query sequences in G1 and G3, or B1 and B3.

>G1_242022
MKYRIFPPVSATLLAAFLPLAADPLDDAILRHRTGVLVIRTAPGTEVTVE
QLRHEFWFGATIATGIFNGRIPAEDAARWKEVFLSHFNAGVIEAAFKWHE
MEKERGKVDYSIVDSMLEWASANGIPVRGHCIFWGIPRYVPEWLKPLGDA
PLRLAVRQRARSIAARYRGQFAEYDLNNEMIHGNWFEERLGPEITRDMAR
WVKEADPEAVLFFNDYDITTGRRLDDYVKHIRTVLSSGAPMDGIGVQGHL
HGDSFDEAELRRALDTLAGLRLPVRITEFNFPGQRSKYYQKRDLQLSPEE
EAAKADALRRFFRICFAHPAVTGILMWGFWEGANWIPQSSLYRRDWTPTP
AAGAYRRLVFGEWWTRATVKADSSGLAAVRAFFGTHRVRAGGREETVALS
RREGAKGIDMQ
>G1_74678
MYNSMHGQISGLRYRAAAKNMIYGAATSSSRLRTDTAYRTTIIQECGIIT
PEYEMKWDRMRPTATTYNFTDADYIVNFARQHGILVHGHTLCWHRALPSW
FDTTVNRSNAEQFLRNHIQTVVSRYRGQIYSWDVVNEAIEPRDATPYHLR
NSRWYQYLGENYIDIAFRAAAAADPSALLVYNEYDVEFDDNIRRDAILNL
LRRLKARRVPIHALGIQGHMRAADRAKLNAHAHLFRQFLNDVAALGLQII
ITEFDCDDRSLPASIQQRDAGVAQMYGDYCAVVMNNPAVVGFITWGITDK
YTELNSSAPRADREPQRPLPLDATMQRKSAWYALEWWFMNTFARPQRTTA
VTNTLPEHMLISELECAPNPAIQQARISFHLNQPRYLHIYIVDVQGRIVA
RLAHEELFSQGLHRRVWDIDPLSCSQGLYYCCISSSGNTVSIPIVVVR
>G1_207691
VEDNVKGKYPGFPLTILLAAFFSAAATTLARPSQSATEIRLRDLVQGRLL
LGAAVEARHLDNPAYARTVATHFNCITAGNEMKPDALQREKGKFTFDRAD
RMVDFAHQHNMTVVGHTLLWHSQSPRWLFEDEAGRPLPREEALTNLREHI
TTVLRHFKGRVIGWDVVNEALSDAPEEYLRDTPARRAIGDDYVIQAFRIA
READPEVELYYNDYNIEADYKRAKALRLVGELREAGVRVDALGIQGHWLL
RHPDLTELERGFAELSATGLRLMVTELDIDPLPRRRAGADVAATEVGLDP
YPDGLPSEVDAELARRYGEVFGLMIRYANEGKLSRITLWGVDDGTSWLNY
WPVRGRTNHPLLFDRQFQPKQAFFAVSRSLEQLRPSAP
>G1_35826
MGSINTLIMKLPALLIFIVCSLPLNVYSQIAAGKCKFLGNVINNSVPPTF
NTYWNQVTPENGGKWGVVESTRDVMNWTTLDLAFNHAKNNRFPFRLHTLI
WGNQQPLWIASLPPAEQLEEIIEWFSLAGARYPGTGWPEDVPFFIDVVNE
PINDPPNQGGDGGNYIQALLGPTGSGYDWIIKAFELARQYFPTAKLHINE
YNVINNDSRTTEYLYIINLLKARNLIDGIGIQCHRFEIETAPSSQLTNNL
NRLAETGLPIFITEFDAGNIGGTGTPDDNQQLEVYQRVFPLLWNHPAVHG
ITLWGYIEGQTWQSTAYLLRSNGTERPALTWLKGFVPTTQGGTFCLTTDL
EKERLKFHVYPNPSSSGRFIVESQVNNLFITVYDVHGRILVHRLLSASNP
TELHVSDHQGIYLMEIKEGNKTHYRKLLIR
>G1_276227
MPNWLAEAQVRIESHRMADVALNLTGADGRPLSHAEVSLELTRHAFKLGA
NGFGIGAIPGADLRERYEERFAALLNYATLPFYWGGYEREPGRTDENRLE
VMADWCARHGISAKGHPLVWHEVFPAWAATLDDADVLRRQQERVKRIVSY
FKGRIDIWDVVNEATVSHTFDNPIGRWIAREGDVSCVAQALAWAREANPS
ATLLYNDFNISPAFERLCAGLIERGAPVDVIGIQSHMHKGRWPIERAWTV
CETYARFGLPLHFTELTVLSGRLKAADDNDWHRIHSDWHSTAEGEAAQLD
YGSQLYTLLFSHPAVEAITWWDFSDFGAWQGAPSGLLRKDMTPKPLYDWL
IDAFHRRWTTRATVVADQAGRASLRAFYGDYALTARLPSGDVVRGTGSIH
LAGSTEITVALA
>G3_81360
MLLRGASRSESDLLAEAEERIRQHRRRRRVLLIRDAKGRPVPGAKVRVQQ
TRHEYWFGCNLFLFGRGSGASWESEYRARFIELFNFATLGFYWAMYEPQP
GRPQEERTDQILEWAAKHGIVCKGHPLVWDDRVSSPSWLPESDEELARLV
ETRVRSVVSRFRGRLDVWDVVNEATHLPDRVSRTRMARWGEAIGPVAYTR
RPLEVARAVHPEAVLLVNDYRLDQAYYNLLDAVRDAEGRPLYDAIGLQSH
MHGGPWPLARIWEVCDRFAQLGRPLHFTEITVVSSSQRSPGEPWGPTNPE
AEARQAEYITKFYTMVFAHPATQALVWWDLSDRGAWQGAAAGLLRRDMSP
KPAYERLHELIRRRWWTQAEGIVGTDGEYVVEAFHGRHRITVEAQSGRTV
TLEVDWPVQAPDRMEVRLQ
>B3_107852
MRISSLVAGLLLTALIFGYGQEKFLGNVFRGYMEPPKFREYWNQVTPENA
GKWGSIAISREIWFWGLLDAIYEYAKEQSFIFKFHTLVWGQQQPLWLVDL
SPEEQRAEIERWFKTVAERYPSIDLIDVVNEPLHSSPLYKEALGGEGVSG
WDWVITAFELARTFFPQSKLLINEYGVISNPTEAQSYAHLVQLLKSRGLV
DGVGIQCHAFEMDYVDLATMRVCLEILAATGLPLYVSELDISGDDQTQLR
RYQEKFPVLWEHPNVVGVTLWGYVQGQMWTENAYLLRVDGTERPALTWLM
EYVRRRHE
>B3_142967
MRLIWSFRSRAAVAALALSAALTVCAQPALKDAFEGVFLIGAALNEAQFT
GRDTNAVALIKTHFNSVTAENVLKWQRIHPEPGVYEFELADKFVEFGVQN
GMVIIGHTLVWHHQTPRWVFRNEDGSQVDRDTLLARMREHIHTVVGRYKG
RIKGWDVVNEALDEDGRLRQTPWLRIIGDDYIAKAFEFAHEADPEAELYY
NDFSLENKPKRDGAVALIKRLKAQGIPITGVGLQGHYTLNWPSKKQLAET
IRAFSELGLKVMITELDVDVLPRADAHRGADLMVRYQHDPKLNPYTAGLP

DSVQRKLAKRYADIFDVLVRHSDAVTRVTFWGVTDRDSWLNYWPMPGRTN
HPLLFDRHYQPKPAFYAVLTSAEKAKKSANKTR
>B3_38007
MVAPLKAFFTGVCFTAVFLLRPINLQAGLPESYRKAWSDLGLVARIEKNI
EQYRKQEARLKLLDSQGRPLPNAAVEIYQKSHAFLFGCNAFVLGQMGEKN
GKYEEMYTRLFNFATVPFYWEGTEPTQGELRYQEGGRDIWRRPPPDRYIP
FAKKYGITLKGHPLLWHSLNPPWIPKEPKELKKLYQKRFAQIAQRYAESI
LIWDVVNESLVCPNTYPLYTPEREYVAWAFQEAQRVFRPENILMINEVQS
VSHAGVGEKNAYFLQIRDLLKRGVKIEGIGFQFHFFTGQTLQQHLECNVY
PPDQLLKVYESFCEFGLPLYITEITIPTTIEDGLEVQAEVLRNLYRLWFS
VPLMAGITYWNLADGTAYKGENEALAGLVDKNLDPKPSYLALERLIHHEW
KTCLSTASDQNGIVTFRGFRGKYTVRVKAGQLEREFEIELPPEGPQSEHH
LQVK
>B3_65635
MSSVGGTLLMKRRQWLGGIVGMAIGTGSSSPSTEENAQFFAQAQERIERH
RKTQLHLDLRRKDGKPVTRAEIKGQQKRHHFLFGSNIFMWGRFPDADSEE
KYRQAFAEVLNYATLPFYWAFYEPKRGQPIHDYIVRVAEWCQQNGITCKG
HPLVWDHEASSPSWLPDDLEEVRRLSLQRVRDIVSRFQGLIDIWDVVNEP
TDLTRFNTTMNRLAQKMGAVPFTLEALQEARRAGPKATLLVNDYRTDPAY
YEILSVLKEKQAPFDVVGIQSHMHAGVWKNERIWDVCQTYQKLSLPLHFT
ETTILSGKRKGEGWEESTPEGEEWQAQETERFYTMLFSHPAVTAITWWDF
ADQGAWMDAPAGWLRKDLSPKPVYERMKELIKKRWWSSFAGVTDEQGRLT
ASVFKGEYQVVIKTPSGDVLTKTVTAAADKTTQITLVI
>B3_97305
MPFSKMDLARGTILGIIVGVLLGYFARTGELEGMKTRISNLESQVGQLRK
FAPSLRTLAEKCGIYIGAAVEPDYLTMDYAETLKREFNMVTTENVLKFGP
VHPQPTVYSFAGADRVIEFAEAHGMKVRGHTLVWHSQLPSWITAGRYTRD
EWIQILRDHIMTVVGRYRGRIYAWDVVNEAIDDSGALRNTIWLQNIGPEY
IELAFRWAHEADPNALLFYNDYGAEGLGVKSDAVYNLVKGLLEKGVPIHG
VGLQMHISADSPPSPQSVAANIRRLNNLGLEVHITEMDVRLRTPASQSDL
IKQAEIYRDILDVCLSAEKCTAFVMWGFTDRYSWIPSTFGGYGSALIFDE
RYEPKPAYYYILQKLIEYSSQRNV
>B3_98625
LAIFGGGLGWQAAAGWAAPGPGPKPTDEELLAGAEERIQRHRKGEAVVEV
ISAGRPVSEATVRIQQTRHRFLFGCNIFMWRPMEADRPEKPKAGQPTSAR
KAAPSDTELQRAYRQRFADLFNYATLGFYWWAYEPKPGQPRHEYTEQVAR
WCQQHGIVAKGHPLAWNWAEPRWLPENPEEVLRLQLDRIEDCLKRFAGLV
DYWDVVNEAVEFDRPECRERAPRLTALWAKLGRAEFTKQCLQRARTASAK
AFLLVNDYIVDPRYEKLLEQLKDREGRLPINAIGIQSHQHGGTWSNRQIW
NVCQRFSRFGLPLHFTETTILSGQRGWELPREKWISTPEGEKWQAQEVVR
FYTMLFSHPAVEAITWWDLSDLGAWQGAPAGLVRKDMSPKPAYEELMRLI
KGQWWTRTEGKTDKAGSFAWNGFFGQYRIEAIVDGRQVEKSVELVPGQKN
RFVLELPAS
>B1_109149
VYRRFLVLILTFLILSIFPISASNPLDFSAVLSFEKGSLDNFVPFGRVKI
SSVKDVAFDGEYSLKVENRVSTWDGCEVDFSQSLVPDITYQVSAMVYHTG
KSPQPFQIIAYVKDLMGERFELVGEVIAMPRTWKKLTGEFKLSYYVLLTK
ASFFVVSPNEIGFDYYVDKFQVLGPNKVEVPGLLVNSTFESRTSEGWEPR
GDKVEVLATKEVARTGDYSLFTKGRSRGWHGAQLNVAKRFQPGRSYSISV
WVYQRSGADQKITLTMERKYNVDADTRYDTIVWQKTVPNNTWVELSGSYS
VPAGVTIEKLLLYVESPNATLEFFIDDIRIVDKRATAANPEPEIPSLYSF
YKDYFRIGTAIPYSVLTNSTEAEMVVKHFNSITPENEMKPDAIQPREGEF
TFTKADAYVKFAEENKLQIRGHTLVWHQQTPAWFFVDKEGKPVSKEVLLK
RLETHIKTLVGRYKGKIYAWDVVNEAIDPAQPDGYRRSKWYEIIGPEYIE
KAFIWAHEADPNAKLFYNDYNTEDVKKRQLIYNLVKSLKEKGIPIHGIGI
QGHIRLDWPSVSEMEQTIQLFSTIPGIEIHITELDMSVYKEPGIEYPKPP
RNLMISQAYRYKAIFDMLKKYKDVVKNVTFWGLKDDYSWLTINRGRNDYP
LLFDKDYQAKLSYWALVEPKVLPPLTQQGAIVKGTAVIDGKEDEAYNIAK
PIVITSEGKNIASVKTLWGPRSIFIFAEVYDTTKDTTDNFTVFFDQNNAK
SPYLQIDDVYLTVRRDGKYESNFTPAIRGVSVREIKEGYVVEAEVIIFGT
TLEKGKKIGIDFAVTDKDRAISWSDTSNQQKAHTVRYGTCELEEAGKLAI
AKKGTPKIDGEMDDIWKNTPEYITDSYVQGQKGKVAYAKFRVLWDETSIY
VYAEIYDSLLNKANANPWEQDSFEIFIDEDNSKRLSYDSNDAQYRVNFEN
VQTFGTNASSARFVTATKKTDFGYIVEAQVKMKEVKLEAGKVIGFDVQVN
DADASGSRVGIIAWNEMENINWQNPSSFGNLRLDQ
>G3_88036
VFLCCAAGRLSQATEPPPTRIQIGRGPTPFTRSTGEPFCPWGFNYDRDHR
GRLLEEYWDTEWPTVAADFAEMKRLGANTIRVHLQVDAFFEAPYRPNPRA
LDRLARLLRLAERTGLYLNLTGLGCYRRSRVPLWYEQLDEAGRWHAQTIF
WTSVARVAARSPAVFCYNLMNEPFVPGTPRAQGGWLAGEFGGFSYVQAIT
LDSAGRPRVMVARQWIQALAGAIRSEDPHALITVGLLPETRATDSFSGFD
PAMVAEEVDFLSVHLYPRGQPHDDARQVLETCSALKPVVVEETFPLHLAP
VLMTAFVREHRRFAAGWLTFYWGQPPEELATQTTFEAVLLHDWLVRWQIL
GRCLNP
>G1_235578
VRLAALACAACACGALVTATFARNLPWVGVSPDGRGFVLLPSHQPFVPWG
FNYDRDDAGRLLEDYWERDWPRVASDFREMKALGANVVRVHLQFGRFMDS

PSRPNRRALAQLSRLVRLAERTGLYLDVTGLGCYHKADVPGWYHALDEAG
RWAAQAAFWRAVAERCRRSPAVFCYDLMNEPVVPGEERRAEWLGPALAGK
HYVQFITRERRGRERTEIARAWVRHLVGAIRRVDARHLITVGLVDWSLER
PGRLFSGFAPERIAPEVDFLSVHLYPESGALEASLETLRGFAVGRPVLVE
ETFPLRCSLEEHARFLEASRAVAAGWLQFYWGRTPEEYRSGRTLQDAIMA
GALEAFRTNAPAFRKP
>G1_161459
VAGAVVVCCSLSGAGGAQEQLPGRWSTARAEQWRRKHPWLVGCNFIPSTA
INQLEMWQAETFDVKTIDRELGWAKDLGFTSIRVFLHDLLWQQDQDGFLR
RIDQFLQIADRHRLGVLFVLLDSCWDPHPKLGPQRPPKPHVHNSGWVQSP
GAAIFGDPKRHDELKDYVQGVLRRFRTDRRVHGWDLINEPNNTNVISYGK
LEPPDKPKLALLLLRKVFAWAREVDPEQPLTAAPWEGDWSKPEKMSPINR
FMFENSDVISFHEYGDFAALKRRVAELKRFQRPILCTEYMARPNRSTFDP
HLGYLQEEDVGAYNWGLVAGKTQTQYPWDSWLKRYTAPPPLWFHEIFHPD
GTPYRAEEVNYIKRLTAKARQGG
>B1_52178
MAEEKRYKNIFRDIEPYKAVEEMNPGWNLGNSLDAIPDETSWGNPKTEPY
IFEDIKKAGFNGVRIPVTWIDHVDSNFNVYKDWMDRVEEVVNYALEKDLY
VIINVHHDSWRWLSKEMRQNKEKTIGKLEKLWLQISERFKNYSEKLIFEI
INEPQYEGFQEWEEGEIQNEVNERILKIIRKSGGFNDKRLVVVPPLSTDI
YKGIKYFRPPKDPNIIIGIHYYSPWDFVANWWGRKSWGSFEDIAQMEKDF
KAFYDKFKDYAIIVGEYGVSNGNRPSEWLYFDNLIRITRKYKMATFYWDN
GFDNFDRRRRIWRDEMKIRIIMNGVKDIPNSFLNPGILFIRGDTLIKDET
VNLILNGNQLVDIIYQGKALNKGRDYVLDNDKVILKSDFLKSIKGELGSS
LVLTFRFNRGADYDLEVIFYKNPVLLDKPLVVLKGLSVPINFLIAFNGTR
LCAIKLIREDNGKPVRDSWTPYLRGWDDFEVKDSQVIIKKHVVEKIDGNV
KVIFEFFPENISLETSLRVK
>B3_136450
MKRRDFLRVGLIGLFSSRLEGSLKKSSGGEVSGAQVASPAGQTPLWKKLP
RWRGFNLLEKFMLPWSNKPFVEQDFKWIADWGFDFVRLPMDYRCWTDPKD
PYQLQEKTLAEIDQAVEWGQKYGIHVCINFHRAPGYTVAQPPEKLNLWKD
PEAQKQFEFHWAAFARRYKGIKPEQLSFNLVNEPANVSAEDYAKVAQRAV
AAIRKEDPQRLIIADGRQWGREPVWELVPLEIAQSTRGYEPMEITHYQAG
WVGRRDWPKPTWPLKRGNQTIDRDWLRQDRIEPWKKLAQAGVGVHVGEWG
AFNKTPHDVVLRWMRDCLTLWKEVGWGWALWNFRGSFGILDSERTDVAYE
DFHGHKLDRKMLDLLRQF
>G3_96404
MRQKSPLVLKPPVMLKRLWVLCCAAAVSACSLPPAPPPRVAAPAQPTPMT
SPTTFEVPGRSFVPLDPFEQNRRLGRGVNLGNALEAPREGEWGVVLQAEF
FPLIRSAGFDTVRVPIRWSAHALREPPYTIAPAFFQRVDWVIEHALKHGL
NVVINMHHYEELFQDVGGERERFLALWDQIAARYRNLPADVLFEPLNEPH
GALTPGVWQRLFEDVLAVIRKTNPQRNVIFTGANWGGPDSLRGMKRPDDP
HLIATFHYYHPFAFTHQGAEWVEGSHAWIGTKWEGNGNQKASIDYDFDRV
ARWAKENNIPLWMGEFGSYGKHADVASRERWTRYVARAAEARGISWAYWE
FGAGFGVYDRDRRRWNEPILNALIPK
>G1_318
MIRIDGKHFRDEQGRTLLLRGVNLGGSSKVPYSPDGRTHLREGFFKHREV
SFVGRPFPLAEADEHFARLALWGLDFVRLLVPWEAVEHAGPGQYDEAYLD
YIRAVVQKGHEHGMRFFIDPHQDVWSRFTGGDGAPGWTLEAVGFDMTHFH
ETEAAFTHQIYGDPFPRMIWPTNYNRLACATMFTLFFAGDDFAPKTHIEG
RSAQDYLQGHFIAAMQQVAARLADLPNVIGYDTMNEPHRGYIGWHDLTQH
ESRWRMGLTPTPYQSMLLGVGLAQMVDVWSFSFTGMKLAGQQRVRPNGVR
AWREGADCVWRQNGVWDFDREGKPHLLRADHFARVDGQPRDFAHYLRPFV
NRYAAGIRQVDPKAIIFIESDALGGEKLERWTEHDAPNVAYAPHWYDGLT
LFTKRYLPFLALDTHGFKPVVGTQAVRRSFVQQIKGHVEQGRSFLGDVPV
VIGETGIPYDMNGARAYQTGDFRAQELCYDATMSALEANLVHFTLWNYTA
DNTNARGDGWNGEDLSIFSRDQQRNPKDLNSGGRALRAVVRPYARALAGE
PLRQRFDMYTGIYECAFRPDSSSSATSEFFIPTLQYPQGVRVSLNEGTYE
LDMTKQRLFVRHSAKDVPHFLRLEPQEARRPQGQRERDRRRALLFGLGLW
LIWRLLRRRR
>G1_196051
MKPMRWFVLPVVALIMISFQRGNGAVSDMEWVQVSDDGRSFVLDPSGVKF
VPWGFNYDHDEKGRLIEDYWESEWAKVEEDFREMKQLGANVVRVHLQVGK
FMESPDRPREAALRQLGRLVKLAEQLRLYLDLTGLGCYHKQDVPRWYDAL
TEKGRWDAQARFWEAVAAQCANSPAIFCYDLMNEPVVPGALKQNDWLGPP
FGGKHFVQFITLETGDRPRPAIARQWIQHLTSAIRKHDRRHLITVGLVPW
SLDRPGLTSGFVPQKICDVLDFISVHIYPEEGKLDEAIRTLQGFAVGKPV
LIEETFPLQCSPHALERFIEASEKIAAGWIGFYWGKTPEECRQSPAIGDA
LMLAWLELFMRKRR
>B1_39173
MKRLYPLSVLFFTLFYCNGKNKIESEKFWQNKIYIYKGEEKILLRGVNIA
HTAKRTIIPWVQREDIERLRKFGLNFIRLTLFWSAVEPERGKYDKNYIEK
IREILRWVDGLGIYVLIDLHQDIYGFHATHSNEGDGPPEWARDPECPPFK
DLDPWMLNYLDESVSCQFESFWNDKNGVQTAYINMFEYVVQQLHNEKGVI
GFEIINEPWPGYEWNDTEKWENEKLREFYNKVIKITRKYTQKLIFYETHP
LSDFGYPFYLQKPDGNNLVYAPHIYPISAFLGSQSQTDTLSIFNKHLEHS
LKYEVPMLVGEYGVAWEDPEAYEKVKEQIDIFDSNFVGSAVWSYDKSSKY

DLAIIDDFGNPLSNFVPVIRPYPEFLKGEIKNIKYLQNELTFEYEGDYFV
LRCPKILDCFIDSEKVSEEQIKISKKKVSVSWRWKDTKE
>B1_46791
MLHIKDSKIVDINTNKEVKLRGVNLGGWLMMEGYILGGRNIPESEFKRRF
KKVNGEAALKEFEYYFRKNFINQKDIEYIKKLGFNCVRIPFHWKIADEEI
IWLDEVIKWCEQNKIYCILDMHSVPGSQNGDWHSDPVKISKSLFWDKKNF
HKKYYELWDLISKRYKDKEIIAGYDIMNEPVIREKNWAKILAGVYNNVIE
IIRKNSDNHIIFVEGNLWATQYDFLNYLDFKNHKNIAISIHFYYPVDFTF
NLDHSLKYPSKILNKYKLHSILKKYLEISKKFNMPIYVGEFGVNLRCYNS
CYGEKQYLKDVISIFEELGYHWTIWTYKTIYINVQPSGLLLYEKNPPFIS
RQENEFGWERYILDWKKYSKQIKTSWLSKNFSFGLIKVLLEIFNKPLDKK
I
>B1_135886
MRVVVSVIVLLTTVTLAFAISPLAVRNGKLVNAKTGKPIGLFGVNLFETH
LGWAVRQDVAEMERNLEAIAKCGFNAIRVPMNMSYIEPAPNVFPDDAQYS
EIMHKHRLKDGFPKFLDALVKKAGELGLYVIFEFHELPSDPWRYFAGGNE
QLRKTGKHGGAISWMANLTLREDGTIEKVELDWEKAIEHVPKALAWLARR
YKGNPIVAAIEVPWNEPIGGLANEEESYFKLVKACAAAVKGVDKNRLVFM
DTQDWGACVNFLPPSSCWRVPDEVDALFPHFYFGMHCPNMPFEISLNAAA
ANWVSWFLAYGKPVLVGEYGIAGLDWKSWLERNKEVLQKRYQLFGEKPMI
GAFYADVLRACLEQWLKMGVQGVFYWAWWEGIPGMAAPFRERTYALTHGH
EVLMEFAPKFQNISITSADAKVVVICEKERRAQYGDPRDLAAISNLLIAA
NVVPYHVIFVEALLGDQRWLRQLKRYQKVIILADGLREGCIELVGKFVEK
GRTFIVRQGEANWQINLQNWLKNL
>B3_100547
MGNQTGWPGAVAWWALAGILGGGFCVGPARPNLAAGAECRSAEQNSAKAV
ARGLEPIRRSPDGRHFVLAQSGKRFLAWGFNYDHDEAGRLLEDYWQTEWD
RVVEDFQQMKQLGANLVRVHLQVGRFLDGPDRPNQRSLEQLARLVQLAEQ
TGLYLDITGLGCYHKKEVPGWYDQLPEPHRWRVQGLFWEAVARTCARSPA
VFCYDLMNEPVLPAPGKKETDWLLGEFGGKYFVQRISLDLAGRKPEQVAR
AWVDNLVDAIRKHDKEHLITVGEIPWATVFPGARSLFHTKEVGDRLDFVS
VHFYPEKGQVQKALKALAVYQVGKPLVIEEMFPLRCSLEELDAFVEGSRE
LADGWIGFFWGKMPAEYAHQKENPFQAALMRSWLEYFQSKSAKMARP
>B3_153879
MIFSFFIFFINAFSQTPVQRHGQLRVSGKNLVDQNGNLVILRGMSLFWSC
WAEGSKYYNSQVINWLVTDWKISVIRAAMGIEPQGGYLSDPTTNKQRVET
VINAAIQNGIYVIIDWHDHNAPRNQAQAISFFQEMAQKYGNYPNVIYEIF
NEPDNDDSWDGIKNYATAVINAIRQRDPDNVILVGTPNWCQDVDTAANSP
ISGVSNVMYVLHFYAASHKDQYRNKAQYAISRNLPIFVSEFGTCEYTGSG
TLDLASTETWLNFLEQNGISWVNWSISDKAETSAALQPGASTSGGWSESQ
LTESGRWIRNKIRSLNTYTSNTVMLYLTSCSSGSIVWATTNVVVNATSQV
GISKVDFYLDGNLVFSDNSSPYIWSWNTQNSLDGWHNLKVVAYTAQNTSA
EATTSVIVYNASAPSVNIVNLQNGATIYGTSVAVYVDANKGYEQTINKVE
LYLNNNKISEDTVAPFSFSIDTTQYSDGNATLRAVAYDKRNQQATKQITV
YIKNQDDPPTVRITYPQNNTTIGGTVNIQVEATDDRSISKVEFYIDGTRQ
FTDYGSPYSWNWDTTQYSEGQHSILVIAYDSSNKQAQASITVVVSRSVQQ
DNPPSVSFVSPTNNQTISGVVTVEVFASDDKGVTKVELYINTTKIAELTA
YPYRFTLNTQNYQNGSYTLKAVAIDTANQFSEVSIVVNINNQTTPPPQDT
PPSVRISSPQNNQTVDKILNLVVNLSDDKGIISRLQIYLNNNRIAEYTSV
NLGTNSYSFDVSSYNNDVYELKVVAIDSSNNSSEAKVNIIINNTFTPPNG
GNDGSTSTESLILDTTILENTTISGIFNFNISAYAKDGISKIEISINNKP
HEVLLFDIPTNNINLNYSLETTSIPDGETQIKITLYDTLNNTTERSYKVQ
VDNFKDHYICTPNNDGLNDYIHFDANSEVRIYNLKGKIIRSIKVSPKIWD
GNDEEGQKVSTGLYIYKITDGNKILAVGTISVIN
>B3_29214
MTLLLILWVILAVVAGCAQVPEGWKFPTATPTPANDPFVQNARLGRGVNL
GNALEAPYEGAWGLVLREEYFRLIKEAGFHSVRIPIRWSAHALEKEPYTI
DSRFFERVDWAVNQALSQGLLVVINMHHFGELMASPQGQRERFLALWKQI
AEHYQGYPPELLFELLNEPSERLTAAIWNELLRDALRVIRASNPDRNVIV
GPVQWNNIRALSSLKLPSEDRHLIVTIHYYEPFQFTHQGAEWVAGARAWL
GTTWQGTEAERRAIEADFDLAATWAKREGRPLYLGEFGAYSTADMDSRAR
WTSFVARQAEARGMSWAYWEFGAGFGVYDRARGDWHKPLLEALIPPDDE
>B3_106662
MEAKNFTNFRPARYAVDYGAKPNLEHLSQAELAIVQPVLSPQEIRLLREG
GTEVLAYISIGEAEPDDPALPNLSSCILGQNPNWGSWYMDARCSEWQDFV
LERARSLREKGYSGLFLDTVDTAELYPQTSPGFVELIRRLRQEIGGIIVQ
NRGFSVLDEVVNYIDGIMHEDLSAGYDFSTGHYLYHEQDPTPALTYRDRL
ILLALDYAPPDRPHLAWKACERARLLGFTGYVAFHVYLHEGGIFCDEVPL
PPSYRATPQGVLRGSEMINLYGLNWFGMETRDRAPHGLWTGRKVPEFLKQ
IRELGFTALRLPLSPQVLWPGYDTASWAQNPGGYPSDAYAGLLYFLEEAR
KEGLYVLLDFHTYDPNRLGASLPGRPFGNGYTKENWLADLRRMAEIALSF
PNVMGIDLCNEPYALTWAEWKALAKEGAETILQANPFILAIVEGVGNMSD
NGGWPAFWGENLTEAYEDPIIKEWHVFLPLVSRAGGSSGLPSRTVTPFLL
GRILYLPHVYGPDVAHQPYFDDPDFPHNLPDIWEIHFGRMAGKFPLGIGE
FGGRYEGKDKIWQDAFVDYLLAKKIHIFFYWALNPNSGDTGGLLLDDWRT
VHEGKLTLLRRLMHK
>B3_230401

MEWKFVEPSGGHFFVELTGKSDVVRVAHSRYFVLNNVWGADTPQTIRVDV
RNGDFTIVHAEHSARNRVAAYPAIIKGNHWELSTTSSGMPIKVGKLISLL
SSWSFTPVDSGRWSAAYDLWFSPLSDSKAGYPGGTELMLWLDRRGAVPDG
SPVTLVTINDIEWEIWFEKGKREWAYVVYVSSRPVYHVDNFNLLEFVMDC
VARGWIEKDWYLHAVEAGFELWEGGVGLASSNFSVLLG
>G1_45801
LRGEAQNAPVSISVDVRANRRAISPLIYGVAHATTEVLADLNCPLNRHGG
NHTSRYNWQLNADNRGNDWYFQSIPYPSATPGEVGDTFIASTRSAGAQPM
LTIPMLDWVARLGPNRTKLCSFSIAKYGPQTDNDWRWFPDAGNGISLLTG
QFITGNDPNDANVRVDASFQQAWVHHLIQRWGTAANGGLRYYILDNEPSI
WHSTHRDVHPVGATMQEVRDRIIEYASRIKQLDPHALIVAPEEWGWSGYF
FSGYDQQWGSQHGWHYLPDRAAQGGWDYLPWLLDQLRQHHTRTGQRLLDI
FSVHFYPQGGEFSENTSTAMQLRRNRSTRALWDPNYTDETWIAAPVRLIP
RLREWVHTYYPGTRIALTEYNWGAEGHINGALAQADLFGIFGREGLDLAT
RWTTPDPSTPTYKAMKMYRNYDGNRSTFGDTSVAATGPDPDNVATYAAVR
STDNALTVMIICKHLSGSTPVTVNLSHFAHRGTAQVWQLTAANVIQRLPD
ISFSGNAFQLTVPAQSLTLLVLPSGNAVAPSFSSTAVVKPATVEPGATAT
ITATFVNTGAPLTNGIIDLEIYNAAGQKVAQRFWTGQHLAMGQKRSFTYS
WTAPQETGAYAVRLGVFGAGWTPLYHWNHHAASIHVGNQDPAQYHFETGL
QGWTHNGGIISSLLRTTERAYAGNYALAVHFKSRRAGQANAFVPLPPTPA
GRTITFRIWFPSGSTITAIQPYVLQGAAGNWRWTGNWQSTASLRAGAWNT
ITVTVPSNAATPLYQLGVEFTTSAPWNGTCYIDSIGW
>G3_120342
MAEKFLFKFPEKFLWGASTSSHQVEGFNYNNWTEWEKNNAYKLSFNAKNY
YQKWQQENFKEMFSPQNYVSGKSTDHYHKFELDFKLAKELGHNSTRFSIE
WSRIEPEEGKFNQKEINHYKQVIELLEKLNLEPFVTIWHWTLPIWFESLG
GFENSKNIFYFKRYVEKIVKNIGKNVKFWITINEPEIYSMMSYLEGKWPP
QKKNIFKYLKVFHNLIFAHKETYKIIKKINPKAQIGIAKNNIYFESYKNK
LANLILKKLADWWWNNYFLNKIQNYQDFIGLNHYFHNRINYGFNKNENKI
VSDLGWEVLPESIYYVLKDLKKFNKPIYITENGLADAQDKKRIWFIFETL
KNIYKALNEGVDIKGYFHWALIDNFEWSDGFWPRFGLIEINYENLNRNIR
LSALFYKEICLNNGITEDIFNKYKNLIDL
>G1_109878
VLPDGPVRPGAEDRVEEGRVFPKGFLWGAATAAHQVEGDNRASDWWAAEE
AGLLPHRSGDACRHRELYERDFDLARSFGHNAHRLSLEWSRLEPEPGRFE
DRWFDHYARVLDALRLRGLEPVVTLQHFTLPRWLASRGGWLARDAVERFA
DYAGEVARRLGRAVRWWITVNEPSVLAKQGYVTGEWPPFRRGRWDLASRV
IARMAAAHRIAHARLHAALPAPMVSFAHSTPWIEPCDPSRRLDRLAARLR
RAFLVDLSFALVRRGGRPLIDYVAINYYTRTVVRWAPRGKALLFGVECRE
PHHGGPRRFDDLGQEVWPEALLHVLRDAARFALPVLITENGIATSDERLR
EEHLRGHLAALARAIEEGIDLRGYLYWSLMDNFEWAEGTTVRFGLAETDF
ATQERRPRRTMRLLAEVARTGRLPSETLPVEV
>G1_188803
MRTSLVLACLLAGGCSAVDRPLPPGFLLGVATAGFQNDPGCPSLPSELCI
DRHSDWYDFVTSEEIRADAGAYVRGDPLEAGPGSYELYSLDFDLVARQLR
GNAVRLSLEWSRLFPQPTDEAEGYEAMRQRADPRALRHYHDVFAALRLRG
LRPLVTLHHYTLPSWIHDAVGCHRDLDRCRRRGWLDRVRIVREIAKYAAF
CGREFGAEVDLWATLNEPFAVVLPGFLFPSWERSNPPAVALRFDEARTAM
VAMLEAHARMYEAVHQSDTIDSDGDGQAARVGLVYNLTPAAPRDPDRPLD
VQAAKRLFYLYNEVFLNAVIRGDLDEDMDGTAVHREDLGGRLDYLGLNYY
TQVIVDGTETPALPRLSPLTTFNPLTLTLWTDHPRGLYDMALLVHSWGLP
VLVTENGTADQSDESVAPRFLVRHLDWLLRAHEAGARIEGYFYWTLVDNY
EWNHGMSMRFGLYRLEQGPDKRRSPREAARVFSEIGAALALPASLRQRYG
GD
>G1_120365
MPEAIHHFPKDFKWGTATAAHQVEGNNTNNQWAVWEQTPGNIHGGDRAGL
ACDWWRNAEADFDRMVELSLNAHRLSVEWSRIEPREGYFDDAAIDRYRQM
LLGLRRRGIEPMVTLHHFTNPIWLEEQGAWENVQVVPKFERFVIHVVRSL
GDLCDVWCTINEPNIYAVMGYLTDDGHMPPGRPGNFDLTLRVIRNMLLAH
AAAYRALHNVQPLARVGLAHHMREFQPLRPNHVLDHLVNRLQDNAFNQGI
LDALMHGRWGLILGRGAEQSARAVRGTLDWIGLNYYSRTRAYFDPRQRST
LFGAIIDTPGGEMSDYDYGEIYPDGLLHCLRRLARFKLPIYVTENGLPDE
DDDQRPAFIVRHLRVLWAAIQFNYKIQGYYHWSLIDNFEWGQGWRMKFGL
YALNPHTQERTPRRSAFLYRDIAKVNALSSDIVREYTPELLPTLFP
>G1_132388
MSVAMADLRFPPGFLWGAATAPVQNEGSYRESAWWTWEQRPGAISHGDRS
GVGCDWWANAEADFDLAHRLGLNAMRLGVEWSRIEPDEGRIDDAALQRYR
QMLQGLRARGMEPMVTLHHFSDPLWFVRRGGWLSHQAPAFFRRFADLVAD
ALGDLVALWVTVNEPMVYYVRDGFLEGRRPPGHRGLLSALVAARNLLLGHA
EAARALRRRRPGAHVGIANHLLHFDPHDPRRSGDRLGARLLDWMFNGWPL
AAQTSGKIKPPYGLGLRTHTLLANSVDFIGLNYYSRVRVRLTTGGHTDFL
VWTLVPLDPSAARFDPGAEGFMGEVYPEGMYRALRRLAAFGKPIYITENG
IADRADRLRGRFIVDHLVQVHRAIAEGVDVRGYFHWTLVDNFEWTDGWQL
RFGLVEMDPATQARRPRPSAAIYAAICRANAIPADLLAEPR
>G3_17268
MRAFPSEFVWGTATAAYQIEGAVAEDGRGESIWDRFAHTPGTIANNETAD
IACDHYHRWQTDIALMRALGVNAYRFSIAWPRILPSGRGAVNERGIAFYD

RLVDGLLEAGITPWVTLYHWDLPQALEDEGGWPNRETAAAFADYAEIVAR
RLGDRVKHWITLNEPWCSSILGYERGEHAPGKRSTPLALRAAHTLLLGHG
MAARALRALWPEARVGITHILTPAYPASDAPLDLAAAARYDAAFNRWFLD
PVLGRGYPGEMLAWWRHAVPRIASSDLDLIAAPIDFLGVNHYFPAIVAHD
PADPYLRLRRITPPGAEVTAMGWVVQPRAFYDLLTRLHREYGPLDLVVTE
NGAAFPDPPPVAGRVADPARVRYLAGYLAEAARAIADGVPLRGYFVWSLL
DNFEWAEGFAKRFGLYYTDYATQERTLKDSGRYYRAVIQAGAPLPADLPL
P
>G3_65204
MPAPPPYVFPRDFLFGAATAGHQIERAQPSDWTAFEQEVMRTGRFGTLGT
GRPKPGHIHDLGRWPDEVRRAKTNHDALYAQLDALAAQMGHNAYRFSLEW
ARLFPKEQMEEADPQGVAYYHGLLDAMERAGLTPSATLFHFASPQWLWQE
RGGRRGWERDDALRHFERYLRAVAKHFGGRISHYCTLNEPMVYVYNGYLE
GVFPPGERRGDPSQVAGVVAQLLRAHALAYRILKEDASRRGRSIAVGFTQ
HTRAFHPYRNWAPLDRLTASLIEQAFIWDFVDAVHTGTLRMTGTQQRQEI
PGLKGTQDYLGINYYGRFYVKTSILHPLRFEILMHDPDDPSEPRSDLGWA
EYPRGLLEVLAEAQRRYQLPLYVLENGVADAADDDRRRQRYLVAHLRELW
LARQRGIDVRGYFHWSLIDNFEWAEGFGPRFGLIRVDYQDGFRRVPRPSA
DLYRRIIRTGITHEMLQAVGLASDR
>B1_72511
MFLVLLIFLFLIVSCASENKNKQKFLWGVAISGFQADMGPNSEPDKNSDW
WIWTHNEENFKKGYYKDVPENSSDFWTHYKADIDIAKDVIGVDVFRYGME
WSRVFPNSTTSVKVSFRTNEIGYPLITIDEKTISELDNIADQKSVKKYRE
ILAYMKQKGLKIFITLNHFSLPYWIHDPIACRDWISGITINAEFSPEQAC
FGKPSGWISSQTVVEFAKYVAYVVSKFGDLVDMWGPINEPIVVATGGYLF
GGISGPLGFGAFPPAGSNKQSFEYVLKNLIFAHAIAYDTIHKFDKEDADK
DGVPTEVFLIYNISYIDGDDQRAVDSAWKLLVWDYLDAVLRGKINSQFVR
ELYKKADIIGINYYNRFQVIYVPVFPSVGMFFAPKPCPSEEPSDVCPYGR
SEMGYELYPPGIYEVIKAVWERYRDLNIPILITENGLSDSQDKKRGIFIQ
EHLRYVGKALDEGIPVLGYFYWSLIDNLEWALGFEKRFGLVFVDFSSPER
KRTIKSSAYVFSDEIKNWRKK
>B3_25863
VERDFPEDFLWGAATAAHQVEGFNVASDWWAAEEAGLLPHRSGAACRHLE
RFEEDFELARRYGHNAHRLSLEWARLEPRPGVFDAEAFAHYARVLAALRA
RGLEPIVTLQHFTLPRWLAERGGWLASDAIERFARYVAEVARALGSGIRW
WLTVNEPTVLAKHGYVTGDWPPFRRGRWDLAMRVIARMIAAHESAYRVLH
EILARPLVSFAHSLPWIEPCDPGRWRDRAAAALRRWFLCDSCFWLVRERG
RPLLDFVAVNYYTRSIVRWRARGRALLFGVDCLDPHHGDRRRFDELGQEV
WPEGLYRTLCRCARLGLPILVTENGIATTDDGLRIEHLSEHLRALARARA
EGVDVRGYLYWTLFDNYEWARGFTAHFGLAALDPENGERRPRPAMELFAR
VARSGRLPRTEEAGEAA
>B3_43607
MITETITFPSDFLWGTATSAHQVEGDNRNNQWWAWEQQPGRIWRGDRSGG
ACGWWMPGGAEADLDLAAEMGQKAHRLSVEWSRIEPSEGMFDRQAIARYR
QILQAMRERGIEPMVTLHHFTDPLWVAHSGGWENPGIIERFRRFVRYTVG
ELGDLVRMWCTINEPNIYVAFGYIRGVFPPGRRHPIRALQVLNHMLRAHA
AAYRTIHQMDGQAQVGLAHHIAVFEPADSSSALDRLMATVHDHVFNAISL
YAPHDGIIRFPAGTGLVYGPLLDSQDFIGVNYYVHYRVRFDPSRRTQGFA
RYLFPPGAPLTDIKADGEPYSALSPEGFYRALRRAAQLRKPIFITENGCP
DAADRVRPRLLATYLPQVHRALREGADIRGYFHWTLVDNFEWADGWGLRF
GLIELDVPTGQRRIRPSGYFYREVARANALTPEMVARYAPELSAYPTSA
>G1_36974
MYLASSLKKSSFGQEFVWGTATAAAQIEGAFLADGKGLSIWDTFSALRGK
IKQNHHNQVACNFYYNYPNDVRLMKEMNIKHHRFSIAWTRVLPEGVGKPN
QKGLDFYDRLIDFHLECGITPWITLYHWDLPQILENQGGWTNREILNWFA
EYVNLCTRKYGDRVKNWMVLNEPMVFTGAGYFLGVHAPGRKWLNNFLPAC
HHAALAMGVGGRIVRQNVKNAHLGTTFSCSWIEPYRPEKELDKQAAFRVD
ALLNRLFLEPVLGLGYPIQDIPLLKKIEKYFLPEDEKLLPFEFDFIGVQN
YTREIVKYSFLTPYINAEIIPGNKRNVLHTLMNWEVYPESIYHILKKYHA
YPQIKKLIVTENGAAFEDHLHHNEVYDTKRLQFLQEYIYQVFRAKQEGVN
VQGYFVWTFMDNFEWAEGYSPRFGIVYVDFDTQKRIVKESGRWYANFLSE
>G1_213144
LWGAATSAYQIEGSPAADGKVPSIWDVFSHRRRKIARHENADIACDHYRR
WREDLAILAEIGFNAYRFSLSWPRLILANGRPNPKGIEHYRKICEFLLER
NIRPFVTLYHWDLPQWLEDKGGWAYRGIVDYFADYAAVVAKAFGDLVNDF
IVLNEPMVFLTLGYLLGVHAPGRRSLRKFFAASHHALLAQAEAARALKAE
KSSLRVGTTISATAVYPASGSKRDVQAAERFDAIYNTFYLEPVLGRGYPT
AKFPFLRRIERFVQAGDMERVRFDFSFWGINTYTRQVVRASRLVPIARWR
QVKNPTAAKNCLGWEVFPRGLYDLLKKYAAYPEIRELIVTENGYADEMTP
VAGRVVDHGRIHYHQQYLAELLRAQREGVPVKGYFVWSLLDNFEWAEGFR
PRFGLVYVDYLTGRRFWKDSAFWWRKFLTGQ
>G1_19558
MSQSSPKTFWWGVATSAYQIEGAVREDGRGESIWDRFCRVPGAVERGDSG
DVACDHYHRYAEDIALMAELDVNAYRFSIAWPRIFPEGRGREEPRGIAFY
DRLVDALLEQGIQPFVTLYHWDLPQALEDEGGWRNRHIVDDFARYVDRVS
RALGDRVKYWTTHNEPWCIATLGYSNGIHAPGVRDPAAALAAAHHLLLSH
GRAVPILRANVPGAKVGIVLNQLWVIPASARAEDAEAARALDGDFNRWYL

DPLFRRAYPEDRVAAYRRDGRLPAGPLPFVHAGDFEEISRPIDFLGVNYY
TCARVAAGDAPGEIRSLPPEGEPTEMGWEVYPQGLYETLMRITRDYAPTS
MLVTENGASYGDGPDENRRVKDQRRIDYLASHIDACERAKRDGAPLDGYF
VWSLLDNFEWQCGYRQRFGIVWVDWSTLQRIPKDSAFWYRDRIRRGLGRD
>G1_112200
MSIQFPANFLWGVATSAYQIEGAWNEDGRGESIWDRFCRRPGNILNGENG
DLACDHYHRWRDDVALMQSLGLKAYRFSIAWTRVLPEGCGNVNAKGLDFY
DRLVDALLAAGIAPNVTLNHWDLPQALQDLGGWPARDCAYRFADYAAVVF
KRLGDRVRFWVTHNEPWCIAFLGYGNGHHAPGICDTSQAYQTVHHLLLGH
GLAVQAFRQGGYSGQIGIALNPQHYVAFSDREADVAARDRYYANAVSLFL
DPIYYGRYPQSLMDWIGPHAPKMQAGDLDLIRRPLDFLGVNYYTTEVISH
SVEGGVLKAHAQPLSAPGWSRTEMGWGVNPPGLTAVLLDLKTKYGNPALY
ITENGCAFSDRPDGQGFCADTARINFLRDHIRAAHAALQAGVDLRGYYVW
SLLDNFEWAWGYSRRFGLVRVDYETQRRIPKQSAHWYREVIARNGLAD
>G1_38
MRFPRDFLWGAATSSYQIEGAVQEDGRGPSIWDIFCATPGKVYGGHSGAI
ACDHYHRYAEDIALMQSLGLQAYRFSVAWPRIFPQGRGKLNPAGLSFYER
LVDGLLAANIAPLLTLYHWDLPQALQDQGGWANRDTLMAFADYADAVARR
LGDRVKLWATHNEPWCIAILSHALGKHAPGKTDWKLALQVSHNLLVSHGL
ALDALRASASAIQAGIVLNFTWATPGGDRQEDEEAAQRYDGFFNRWFLDP
LFKGSYPQDMWDIYGARVPRIEADDMALIQRPMDFLGVNYYTRELVRPGQ
DGFSFRFERNPKAEYTAMNWEVYPQGLEDMLVRLHEDYSAPPLYVTENGA
AFDDVLTPGGVRDARRRAYLEAHFQAAYNAMQRGAPLRGYFVWSLLDNFE
WAEGYAKRFGIVYVDYKTQKRIPKDSALWYRDFIAQQRA
>G1_210987
MKKSDFGNDFVWGVATAAYQIEGAWNADGKGESIWDRFTHSYNNIERCET
GDLACDHYHKYPEDIALMKQLNIQASRFSIAWTRIFPYGEGKTNPLGVAH
YHKVIDTYLEAGIQPXXXXVTLYHWDLPYILEKRDGGWTNRSTVNYFCEY
VDFVTKEYGDKVKHWMILNEPMVFTGLGYFQGIHAPGKNGIQFLLAAIHH
AALALAEGARIAKSNCPHAEVGSTFSCHLVQPKSKFFLHEGAANRYHAVL
NRMFIDPASGRGYPIDAIPMLKRIEKYFQPGDDKRIIAELDFVGIQNYTR
EIIQFSPFVPFLWGKPVDAKKRGVHTTEMGWEVYPESIYHCLKFFASYPE
VKKIYVTENGAAFPDTVTRSRTIEDYQRIEFLKAYLAQVLRAKREGVNVQ
GYFVWSFMDNFEWNFGYRPRFGLVYVDYPTQERIIKKSGEWYRDFLAGD
>G1_47976
VAQRLQFPHDFVWGVATSSYQIEGAWQEDGKGESIWDRFSHTPGKVLNGD
TGDVACDHYHRYVEDVALMRSLGIGAYRFSIAWPRVQPQGHGPVNPAGLD
FYDRLVDALLAAGIRPFVTLYHWDLPQALQDRGGWGNRDVARWFADYAHI
VARRLGDRVKHWITHNEPWVVAMVGHLIGEHAPGLQDPALAVRVAHHLLL
SHGLALEPIRSESAGCQAGITLNLIPMYPASDKGADLDAAARNDGFVNRW
FLDPLLRGRYPEDMLALWEQIVPEVDPDDLAVISRPIDFLGVNYYTRQVV
KHDQEGNPLLQVALVHPPGEYTTMNWEVYPEGLYELLMRLHREYAVPALY
ITENGAAYPDVVDAEGRVRDPKRVAYLRSHLVQAHRAIADGVPLRGYFAW
SFMDNFEWAHGYSQRFGLVYVDFATQERIVKDSGRWFGEAAQENGVEP
>G1_298950
MSFPKNFLWGAATAAYQVEGAVNEDGRGRTVWDLFCERPGAVWSGHNGAV
ASDHYHRWREDVALMRQLGLRAYRFSIAWSRVLPEGTGRVNPKGLAFYDR
LVDALLAAHIEPFITLFHWDYPAELYYRGGWLNRDSADWFADYAAVVARK
LGDQVRHWLTLNEPQCFIGLGHQDGIHAPGLKLGWTEVLRAAHHTLLAHG
RGVQALRAHCRARPRIGWAPVGMVKLPASNRASDVRAAREATFAVRSGSL
WNNTWWMDPVYLGRYPADGWKAYAPYTPPVRDGDLKTIHSPLDFFAFNIY
HGDFVRAGQPEPLRWPEGNPLTLFYWPVTPPCLYWAVRFFAERYRLPIII
TENGMSGVDWVAEDGRVHDPQRIDFTRRYLRELRRAIAEGVDVRGYFHWT
LLDNFEWAEGYRQRFGLIHVDYPTGRRTPKDSFHWYRQIIATNGDALDTD
PKTLFH
>G1_145459
MSPAWPEGFLWGVSTSAYQIEGATAADGRGPSIWDEWAKVPGRMRDGETG
DPASGHYLRWREDVALLRELGARAYRFSIAWPRVLPLGRGPVNAAGLDFY
DRLTDALCEAGIVPIACLYHWDLPQALQEEGGWPARDIAGRFADYAAISA
ARLADRIPFWATFNEPGLFTMFGHLTGGHPPGLKRTDAYLAAMHHVNLAH
GGATRAILAVRSDARVGCVHNVQPVRPGSDAPADAEAAGMLGELWNRALP
DAQLLGHYPDRIAALMEPWIRGGDLAAIRAPAAWIGVNHYSPVYAKHDPS
LPFACAFTDAPRDGTPATPINWRIEPVAFRDTLTDVHARYRLPVVVTENG
YGAEEDGTTLEDAGRIAFHEAYIGAMREARAQGADIRGYLAWTLLDNLEW
SGGRRVRFGLVRVEPDTQERRKKASFAWFARLFRAAPEG
>G1_149057
MQRRNFLSCLLATTAGLVTNPNQLIGLAATSTLKGSDFGKNFVWGVATAA
YQIEGAASADGKGPSIWDEFTHRKGKVKNNENGDKACEFYYRYKEDLELM
RLLGIPAFRFSIAWSRLFPEGIGRINPAGVDFYHRVIDTCLEKEITPWIT
LYHWDLPLALEKKGGWLNREIIHWFSEYCDFCTKTYGDKVKNWMVLNEPM
AFVGVGYFLGYHAPGRKGIFSFLKATHHATLAQGIGGKVVKANVSGANVG
TTFSCSHIAAYRNDKEKDQIAAMRADALFNRLFLEPALGLGYPIESLPFL
KYIERYFQPGDEEKMKFDFDFVGLQNYTRELVKYSPFVPMLWAKIVPASK
RGIRTTEMGWEIYPEGIYCLLKKFASYPQVKKIYITENGVAFPDTVLLGE
VYDYDRIEFLESYLKQVLRAKREGVPVEGYFIWSFMDNFEWAEGYKPRFG
IVYVDFETQKRIIKQSGYWYRNLIHS
>G1_67084

```
MGPSALRAAIQEDTPLTADQFGPGFLWGTATAAYQIEGAWNKDGKGPSIW
DDFSHKKGHIKTGENGDVACDFYHRYPEDLDLLKQMHFDVFRFSIAWSRI
LPEGTGAPNVAGLAFYDRVIDACLERQIQPWITLYHWDLPLALHHRGGWR
TRDIVGWFSDYVALCARRFGDRVKNWMVLNEPMAFTALGYFLGMHAPGER
GFGKFKRAVHHAVLCQAEGGRILRSEVSNAHIGTTFSVMPVQPKSNKEKN
ILAARRADALFNRLFIEPALGLGYPVDGFPYLRTMEKLMQDGDTEKMKFD
FDFIGLQNYTRFVAGFSLFPPVLWAHQVKPARLAGGREHLTEMGWEVYPE
GMYQILKQFAAYPGVRKIIVTENGAAFPDTIQDDGVHDIRRVRFFQDYLR
QALKAKKEGVPLAGYFVWSLMDNFEWAEGFKPRFGLVYVDFKTQKRILKD
SGLWWKNFLEKKGH
>G1_173352
MRGWIDFPKDFTWGTATAAYQIEGAHNVDGKGESIWDEFCKRKGKIKNGD
IGDVACNHYHQYKEDLNLMKSLGYPAYRFSISWTRIFPEGKGKLNQKGLD
FYKKLVDELLEKNILPFVTLYHWDLPLELEKQGGWLSREVPKYFADYSEV
VVTTFQDRVKHWITLNEPWSVMIGGYVLGILAPGKMRPFQSLRVAHNLLL
AHGLAVERIRNISSSLQVGITHALSPIHPASLNGSHKATFRAHALNNELW
LDPILKAKYPKEIEQQVFSQNKKIPLEEDLKIISQKIDFLGINNYTRTIV
RSFPFPLYSFRPIRPTYPNVQFTSMNWEIYPRGIYEILKWIQENYKNPPV
YVTENGVAFYEKPNESGEILDENRIQFLKSYLSEVSHAIHEGVNVKGYFV
WSFMDNFEWAYGYEKTFGLVHIDRKTLRRTPKKSAFWYSQVCRENGFLY
>G3_101385
LPIAPKDNFLYGVATSAAQIEGAAWEDGRLDSIWDVFARKKGKIKDRSHP
SPGCDHYHLWQKDVENLVWLGVNAYRFSLSWSRLIPLGRGELNPKGVSFY
QRLLAALRKNDIRPYVTLYHWDLPQSLEDKGGWTNRDITSWFVEYAHKVD
KALGEFIDSYIILNEPLVFTALGYLLGIHAPGRRGLANFLKAMHHALLAQ
AEAARAIRAQNKQVRLGTSISCVMGYPYRQQEKDLRALARFDALMNRLFV
DPVVGRGYPTQELPVLKKAERYFAPQDLEKICFDFDFWGINSYTTKLVKY
AWYVPYVHYREIRRDLPRTEMDWEIEPQGIYDLLKKFGSYPEIKEILISE
NGAAFYDEVIGGKIYDFRRINYLKQHLAMVEKARQEGVPVRGYFVWSLMD
NFEWAEGFRMRFGLFYVNYETKERLPKESAWWYRELIRGNT
>B3_80816
LAVLGYTDAVEPTTQLLPPWLWGAATSAFQVEGSPLADGAGPSNWYVFTH
TPGKVRGGHTADVACDHYRRFPEDVGLMRQLGLQAYRFSLAWGRILPEGR
GRINTRGLDFYQRLVDQLLEAGVQPMVTLFHWDLPWELERQGGWANPATV
EAFAHYGQVVFRALGDRVRLWVTLNEPWVVMDAGYVHGVHPPGRREPAVA
VRVAHHLLLAHARAVQVGRAQGVGAVGLVVNLEPKHPGGEREEDRQAAQR
AHTYNNRLFLDPLFFGRYPEDVEEAFGTPWPEELCCQVEQVRGSLDFLGI
NYYTRSVLVHDPQAPPPKVRRLPPLAEASTTMGWEIYPQGLGETLHWVAH
QYPKVPLLVTENGAAFADPPPGGGRVADQRRVRFLREHIQQVLAARAQGV
DVRGYFLWSLLDNFEWTEGYFQRFGVVYVDFASQQRTIKDSGWFYRQVIA
SDGRLLQHSFTFPSGDR
>B3_165627
MRVKEILKKMTIEEKVFQLCSVYITDIVKDGEISNELLEKELEFGIGQVS
RVYGGIKNIDPEKAKEYLEKIQKFLKEKTRLGIPAMIHEECLSGFLTNKA
TSFPQIIGIASSFNPKLIEKMTRVIRRQMRNAGVHQGLSPVLDVCYDVRW
GRTEETFGEDPYLCALMGVHYVKGLQGENLKEGIVATGKHFAGHGFSEGG
RNICPVHVGERELRDIFLFPFEACVKEANLKSIMNAYHDIDGIPCACSKK
LLTDILRKEWGFDGIVVSDYEAIKMLNTIHLVAKDDKECAILSLKAGIDI
ELPNKSCYPLLIECVKKEIIKESEIDKAVERILKIKKELGLFEENKKFFI
DFDKEEDRKLAYEIAKETFVLLKNDGILPLKNIKSISLIGPSVDSPRNYF
GDYAYTAHLNLEKPSVECKSILEVFRERGIEVYYEKGCDIFDFNKENFEK
AIEIGKKGEVIIFVGGDKSGFASDCTCGEGKDSHNLKLPGVQEDLILKLS
ELGKPLIILLITGRPYILTDIIDKVNAIVECWFPGEETANCIFDMLFGKF
SPSGKLPVSFPKHPGQLPVYYHRKPVSLRNRYVYLDIQPLFPFGFGLSYS
SFELFNFKIEQEKIKAGKNFNVFVSIKNTGNIEAMETVQLYIRKKFSSCV
LSTKFLKGFYKVNLRPEEEKTIKFEIPSEVLAFRDENMRLKIEEGKYEVM
IGFSSEDIKYKGEIEIIGNKFLKERKVFFSDVNTI
```

Figure S1. Fasta sequences of novel CAZymes from the shotgun contigs. The primary sequence has ≥90% subject coverage and 50–70% identity to the deposited protein sequences.
